# Hyperoside inhibits PRRSV proliferation via the TLR4/NF-κB and p62-Nrf2-Keap1 signaling pathways, mediating inflammation and autophagy

**DOI:** 10.1128/spectrum.03107-24

**Published:** 2025-06-12

**Authors:** Tongtong Wang, Li Chen, Anqi Wang, Yulin Xu, Yuyu Zhang, Qin Zhao, Qinghai Ren, Sidang Liu, Liangliang Li, Yubao Li, Jiaqiang Wu

**Affiliations:** 1 Key Laboratory of Livestock and Poultry Multiomics of MARA, Shandong Academy of Agricultural Sciences, Institute of Animal Science and Veterinary Medicine 722363 , Jinan, Shandong, China; 2 College of Agriculture and Biology, Liaocheng University 58291, Liaocheng, China; 3 Department of Preventive Veterinary Medicine, College of Veterinary Medicine, Northwest A&F University 12469, Yangling, Shaanxi, China; 4 College of Animal Science and Technology, Shandong Agricultural University 34734, Tai'an, China; Thomas Jefferson University, Philadelphia, Pennsylvania, USA

**Keywords:** hyperoside, PRRSV inhibitor, anti-inflammatory agent, autophagy

## Abstract

Porcine reproductive and respiratory syndrome virus (PRRSV) causes abortion and respiratory disease in swine, hindering the development of the pig farming industry worldwide. However, at present, there are no effective vaccines or drugs for PRRSV control. In this study, we evaluated the inhibitory effect of hyperoside on PRRSV replication *in vitro* and *in vivo* and explored the underlying mechanisms. Our results revealed that hyperoside significantly inhibited PRRSV infection in MARC-145 and porcine alveolar macrophages (PAMs). This inhibition was linked to the hyperoside-induced attenuation of pro-inflammatory cytokine (IL-1β, IL-6, IL-8, and TNF-α) upregulation induced by PRRSV infection, which was mediated by the suppression of the Toll-like receptor 4 (TLR4)/nuclear factor kappa B (NF-kB) signaling pathway. Moreover, hyperoside alleviated the autophagy induced by PRRSV via p62/Nrf2/Keap1 signaling pathway activation. *In vivo*, hyperoside treatment led to an obvious decrease in PRRSV replication in piglets. Therefore, hyperoside may be a useful antiviral agent against PRRSV.

**IMPORTANCE:**

Porcine reproductive and respiratory syndrome virus (PRRSV) causes abortion and respiratory disease in swine, which induces huge economic losses every year. However, there have been no effective vaccines or drugs for PRRSV control until now. Our present study found that the inhibitory effect of hyperoside on PRRSV replication in *vitro* and in *vivo*. Furthermore, we demonstrate that hyperoside inhibits PRRSV proliferation via inhibiting inflammation and autophagy through the Toll-like receptor 4 (TLR4)/nuclear factor kappa B (NF-κB) and p62-Nrf2-Keap1 signaling pathways. Hence, we believe that hyperoside may be a useful antiviral agent to control PRRSV.

## INTRODUCTION

Porcine reproductive and respiratory syndrome virus (PRRSV) is an enveloped, positive-sense single-stranded RNA virus with a genome size of 14.9–15.5 kb, and it belongs to the *Arteriviridae* family (1, 2). This virus causes reproductive disorders in sows and severe respiratory disease in swine, generating massive economic losses in the global pig farming industry (3, 4). Since its emergence in the 1980s, PRRSV has undergone significant mutation and recombination (5, 6). However, effective vaccines or drugs that prevent or control PRRSV have yet to be developed, and there is an urgent need for new effective anti-PRRSV drugs.

Hyperoside is a natural flavonoid that can be extracted from various plants, including *Cuscuta chinensis* Lam., *Forsythia suspensa*, and *Crataegus pinnatifida* Bge (7). Previous studies have shown that hyperoside possesses a broad spectrum of biological activities, including anti-inflammatory, antiviral, antioxidant, antihyperglycemic, and anticancer activities (8–11). In recent years, hyperoside has also been found to inhibit several human and animal viruses, such as herpes simplex virus type 1 (12), hepatitis B virus (13), infectious bronchitis virus (14), severe acute respiratory syndrome coronavirus 2 (SARS-CoV-2) (15), African swine fever virus (16), porcine epidemic diarrhea virus (PEDV) (10), and equine herpesvirus type 8 (EHV-8) (17). However, information regarding the anti-PRRSV activity of hyperoside and the potential molecular mechanisms underlying its effects on this virus is limited.

Both inflammatory cytokines and autophagy play crucial roles in viral infection and pathogenesis (18). PRRSV infection significantly increases the levels of inflammatory cytokines such as IL-1β, IL-6, IL-8, and TNF-α in porcine alveolar macrophages (PAMs) and swine (19, 20), with uncontrolled inflammation aggravating lung damage (21). Recent studies have shown that compounds that downregulate pro-inflammatory factors (22), such as the itaconate derivative 4-OI22 and allicin (23), can effectively inhibit PRRSV infection. Similarly, autophagy—an evolutionarily conserved cellular process that maintains homeostasis (24–26)—can either promote or inhibit viral replication, depending on the virus type (27, 28). Previous research has demonstrated that PRRSV-induced autophagy facilitates viral self-replication (29, 30), while disrupting this autophagy response can suppress PRRSV infection (31). Notably, hyperoside has been shown to inhibit inflammatory responses (32) and suppress autophagy (33, 34) in various disease models, although its effects on PRRSV-induced inflammation and autophagy remain unexplored.

In the present study, the anti-PRRSV activity of hyperoside was investigated, both in susceptible cells and in piglets. Moreover, the potential anti-PRRSV mechanisms of hyperoside were explored. Our results showed that hyperoside could effectively confer resistance against PRRSV *in vitro* at multiple replication stages. Further analysis revealed that hyperoside could also attenuate the overexpression of pro-inflammatory factors induced by PRRSV by suppressing the Toll-like receptor 4 (TLR4)/nuclear factor kappa B (NF-κB) signaling pathway. Additionally, our findings showed that hyperoside alleviated PRRSV-induced autophagy by activating the p62/Nrf2/Keap1 signaling pathway. Finally, we demonstrated that hyperoside was effective in reducing the PRRSV load and lung injury in piglets *in vivo*. Our data indicate that hyperoside may serve as a promising therapeutic agent against PRRSV.

## MATERIALS AND METHODS

### Cells, viruses, and reagents

MARC-145 cells were obtained from the China Center for Type Culture Collection (Wuhan, China) and maintained in Dulbecco’s modified Eagle’s medium (DMEM) supplemented with 10% fetal bovine serum (FBS) and penicillin-streptomycin at 37°C under 5% CO_2_. PAMs were obtained from 8-week-old PRRSV-negative pigs as previously described and cultured in RPMI-1640 medium supplemented with 10% FBS.

The PRRSV-1 strains GZ11-G1 (GenBank: KF001144.1) and P073-3 (GenBank: MK214314.1) and the PRRSV-2 strains SD16 (GenBank: JX087437.1), CH-1a (GenBank: AY032626), JXA1 (GenBank: EF112445.1), and NADC30-like (GenBank: KX766379) were used in this study. These PRRSV isolates were allowed to proliferate in MARC-145 cells and titrated via a plaque formation assay, as described previously (35, 36).

Hyperoside was obtained from Sigma‒Aldrich (Saint Louis, USA) and dissolved in dimethylsulfoxide (DMSO). 3-Methyladenine (3-MA, an autophagy inhibitor) was obtained from Shandong Sparkjade Biotechnology Co., Ltd. (Jinan, China). Finally, porcine PRRSV convalescent serum, which contained monoclonal antibodies against the PRRSV-1 and PRRSV-2 N proteins, was prepared in our laboratory.

### Cytotoxicity assay

The cytotoxicity of hyperoside to MARC-145 and PAM cells was examined as described in previous studies (17). Briefly, MARC-145 and PAMs were preseeded into 96-well plates overnight and then treated with various concentrations of hyperoside (0, 15, 30, 60, 120, and 200 µM) for 24 h. Then, the medium was removed, and the cells were resuspended in 50 µL of DMEM or RPMI-1640 containing 10% Cell Counting Kit-8 (CCK-8; Shandong Sparkjade Biotechnology Co., Ltd., Jinan, China) and incubated at 37°C for 2 h. To calculate cell viability, the optical density (OD) of each well was measured at 450 nm via an Epoch microplate spectrophotometer (Biotek, USA) and analyzed via GraphPad Prism 8.0. DMSO was used as the negative control for these experiments.

### RNA extraction and quantitative real-time polymerase chain reaction

All the cell samples were harvested at the indicated time points, and total RNA was extracted via SparkZol Reagent (Jinan, China); subsequently, 1 µg of RNA extracted from each group of cells was reverse transcribed into cDNA via the PrimeScript RT Master Mix (Takara, Japan). Then, quantitative PCR (qPCR) was performed to quantify target gene expression via the 2^−∆∆Ct^ method, as described previously (37). *GAPDH* transcripts were amplified to normalize the total RNA input. The primer information is listed in Table 1.

**TABLE 1:** List of primers in this study

Genes	Forward primer (5′−3′)	Reverse primer (5′−3′)
IL-6	CAGCCCTGAGAAAGGAGACA	CCAGGCAAGTCTCCTCATTG
IL-8	ACTCCAAACCTATCCACCCC	CCACAACCCTAGACACCCAT
TNF-α	TCCCCAGGAAGACAGCG	GCAGCAGACAGAAGAGCGT
IL-1β(Marc-145)	GGAAGACAAATTGCATGC	CCCAACTGGTACATCAGCAC
IL-1β(PAMs)	ACCTGGACCTTGGTTCTCTG	CATCTGCCTGATGCTCTTGT
GAPDH	GTCTCCTCTGACTTCAACAGCG	ACCACCCTGTTGCTGTAGCCAA
ORF7(PRRSV-1)	ATGGCCGGTAAAAATCAGAGCC	TTAATTCGCACCCTGACTGG
ORF7(PRRSV-2)	ATGCCAAATAACAACGGCAAGC	TCATGCTGAGGGTGATGCTGTG
siTLR4^*a*^	GCAAATGCCTCTGTGATTT	AAATCACAGAGGCATTTGC
siTLR4^*b*^	GTGCAGGCATAATCTTCAT	ATGAAGATTATGCCTGCAC

^*a*^ Swine.

^*b*^ Monkey.

Absolute quantification was also performed to evaluate the RNA genome copy numbers of PRRSV-1 and PRRSV-2 in the cell culture supernatant, as described previously (35). Two recombinant plasmids, pMD18-T-N-1 and pMD18-T-N-2, containing the PRRSV-1 *ORF7* gene and PRRSV-2 *ORF7* gene, respectively, were used. These plasmids served as templates for qPCR at the indicated times.

### Western blot analysis

The cells were collected at the indicated time points and lysed with 2× Laemmli sample buffer. The lysates were then boiled, and equal amounts of samples were separated via 10% SDS-PAGE and transferred onto polyvinylidene fluoride (PVDF) membranes as described previously (38, 39). The PVDF membranes were further blocked with 5% serum albumin and incubated with specific antibodies to detect different target proteins. α-Tubulin served as the loading control. Finally, the membranes were imaged via a ChemiDoc XRS imaging system (Bio-Rad, USA). The primary antibodies used in the present study included antibodies against TLR4, NF-kB p65, p-NF-kB p65, IkB, p-IkB, and LC3 (Cell Signaling Technology, USA); anti-p62 and anti-Nrf2 antibodies (Servicebio, Wuhan, China); and anti-α-Tubulin and anti-Keap1 antibodies (Abcam, Cambridge, UK). Horseradish peroxidase-conjugated anti-mouse IgG and anti-rabbit IgG were purchased from Thermo Fisher Scientific (Massachusetts, USA).

### Viral titration

MARC-145 cells were grown to approximately 80%–90% confluency in 96-well plates. The viral suspensions were serially diluted 10-fold, and 100 µL of each dilution was added to the wells (*n* = 8) and incubated for 1 h. Subsequently, the cells were incubated with DMEM containing 3% FBS for 5 days. The 50% tissue culture infectious dose (TCID_50_) was calculated via the Reed-Muench method according to previous reports (36) and analyzed via GraphPad Prism 8.0.

### Antiviral activity assay

MARC-145 cells and PAMs were seeded into 12-well plates and cultured until they reached 90% confluence. These cells were subsequently treated with various concentrations of hyperoside (15, 30, 60, or 120 µM) or the indicated medium containing 0.1% DMSO for 2 h. Following incubation, the cells were infected with PRRSV SD16 for 1 h at a 0.1 multiplicity of infection (MOI). The medium was subsequently replaced with fresh DMEM or RPMI-1640 containing 3% FBS and the indicated concentration of hyperoside. These cellular supernatants and cells were collected at 24 hours post-infection (hpi) for further qPCR and western blot analysis.

### Time-of-addition analysis

To further determine which stage of the PRRSV life cycle was disrupted by hyperoside, MARC-145 cells were seeded into 12-well plates and divided into pretreated, cotreated, and post-treated groups according to the timing of the 120 µM hyperoside treatment relative to PRRSV SD16 (MOI = 0.1) inoculation. Specifically, the pretreated group was incubated with 120 µM hyperoside for 2 h and then infected with PRRSV SD16 (MOI = 0.1). Moreover, the cotreated group was treated with a mixture of hyperoside (120 µM) and PRRSV SD16 (MOI = 0.1) for 1 h. Finally, the post-treated group was infected with PRRSV SD16 (MOI = 0.1) for 1 h, and the medium was then replaced with 3% FBS DMEM containing 120 µM hyperoside. After 24 h, qPCR and western blot assays were performed to examine PRRSV replication in these groups.

To further test whether hyperoside interacts with PRRSV directly, a virucidal activity assay was performed as follows. A mixture of hyperoside (120 µM) and PRRSV SD16 (0.1 and 1 MOI) was incubated together at 37°C for 2 h. Then, MARC-145 cells were treated with this mixture at 37°C for another 1 h. At 24 hpi, the cells and cellular supernatants were collected to assess PRRSV replication via western blot and qPCR assays.

### Viral binding, internalization, replication, and release assays

For the binding assay, MARC-145 cells were cultured in 12-well plates and preincubated at 4°C for 1 h. Then, the cells were inoculated with PRRSV SD16 (MOI = 1) and hyperoside (120 µM) or 0.1% DMSO and incubated at 4°C for 2 h to facilitate viral attachment. These cells were then washed three times with prechilled phosphate-buffered saline (PBS) and harvested to extract viral RNA for qPCR quantification.

For the internalization assay, MARC-145 cells were seeded into 12-well plates overnight. These cells were then pretreated with hyperoside (120 µM) for 2 h before infection with PRRSV SD16 (MOI = 1) at 4°C for 1 h to allow viral attachment. The cells were then washed with ice-cold PBS to remove the unbound virus and incubated with 3% FBS DMEM and 120 µM hyperoside or DMSO at 37°C for 1 h. The cells were treated with citrate buffer (pH 3.0) to remove any noninternalized virus particles. Finally, viral RNA was extracted and quantified via qPCR.

For the viral replication assay, MARC-145 cells were cultured in 6-well plates and infected with PRRSV SD16 (MOI = 1) for 6 h. These cells were washed with PBS and then incubated with 3% FBS DMEM containing hyperoside (120 µM) or DMSO at 37°C. The cells were collected at 7, 8, 10, and 16 hpi to quantify PRRSV genome copies via qPCR.

For the viral release assay, MARC-145 cells were cultured in 12-well plates and infected with PRRSV SD16 (MOI = 1) for 24 h. The cells were washed with PBS and incubated with 3% FBS DMEM containing hyperoside (120 µM) or DMSO for 30 or 60 min at 37°C. Finally, the cell supernatants were collected to detect progeny virus particles via qPCR.

### Small-interfering RNA assays

The siRNA knockdown assay was performed as described previously. MARC-145 cells were transfected with siRNAs targeting Nrf2 or the siRNA negative control (siNC) for 12 h and then treated with hyperoside (120 µM) for 2 h. Subsequently, the cells were infected with PRRSV SD16 (MOI = 0.5) and harvested at 24 hpi to analyze the protein expression of p62, Nrf2, Keap1, LC3I, LC3II, and PRRSV N via western blotting. MARC-145 cells or PAMs were transfected with siTLR4 or the siRNA negative control (siNC) for 12 h and then treated with hyperoside (120 µM) for 2 h. Subsequently, the cells were infected with PRRSV SD16 (MOI = 0.1) for 24 h. The levels of IL-6, IL-8, IL-1β, and TNF-α mRNA in the cells were analyzed via qPCR, and the production of IL-6, IL-8, IL-1β, and TNF-α in the supernatants from the PAM cultures was analyzed via enzyme-linked immunosorbent assay (ELISA).

### ELISA

The supernatants from the PAM cultures were harvested. IL-6, IL-8, IL-1β, and TNF-α production in the supernatants was analyzed via porcine IL-6, IL-8, IL-1β, and TNF-α ELISA kits (Jianglai, Shanghai, China) following the manufacturer’s instructions.

### mCherry-GFP-LC3B puncta assay

A mCherry-GFP-LC3B puncta assay was performed to detect the degree of autophagy induced by PRRSV infection, as described in previous studies (40). Briefly, MARC-145 cells were seeded on coverslips and placed in 12-well plates. Then, these cells were transfected with the mCherry-GFP-LC3 plasmid (Miaoling plasmid, Wuhan, China) for 12 h, treated with different concentrations of hyperoside (60 µM and 120 µM) or 3-MA (30 µM), and infected with PRRSV SD16 (MOI = 0.5) for 24 h. The cells were fixed with 4% paraformaldehyde and stained with DAPI (Invitrogen, USA) for 15 min. Finally, the cells were observed and imaged via an inverted fluorescence microscope (DMi8, Leica, Germany).

### Animal experiments

Sixteen 6-week-old PRRSV-negative piglets purchased from Xilingjiao Science and Technology Ltd. (Jinan, China) were randomly divided into four groups (four piglets/group). Group 1 was infected with PRRSV SD16 only (3 × 10^5^ TCID_50_/piglet), while Group 2 was treated with hyperoside (75 mg/kg) and then challenged with PRRSV SD16 (3 × 10^5^ TCID_50_/piglet). Group 3 was treated with hyperoside (150 mg/kg) and challenged with PRRSV SD16 (3 × 10^5^ TCID_50_/piglet). Finally, Group 4 served as the negative control group (mock group). At −1, 1, 3, 5, and 8 days post-challenge (DPC), piglets from Group 2 and Group 3 received the same oral dose of hyperoside. Moreover, piglets from Group 1 and Group 4 received an equal volume of MEM orally (including 0.1% DMSO). Piglets from each group were housed in separate rooms to prevent cross-infection. Clinical symptoms and rectal temperature were monitored daily.

All the piglets were euthanized, and their lungs were collected for pathological analysis and immunohistochemistry examinations at 28 DPC. Necropsy and pathological analysis were performed as described in our previous study (36), and pathological scores were calculated. The PRRSV loads in the serum were detected at 4, 7, 10, 14, 21, and 28 DPC, as described previously (41).

### Statistical analysis

The data are presented as the means ± SDs and were analyzed with GraphPad Prism 8.0 software. The intensities of the western blot bands were analyzed via ImageJ 1.8.0 software (National Institutes of Health). Differences among the groups were analyzed via the unpaired Student’s *t*-test. Statistically significant and very significant differences were defined as those with *P* < 0.05 and *P* < 0.01, respectively.

## RESULTS

### Hyperoside suppresses PRRSV infection in various susceptible cells

**Fig 1:**
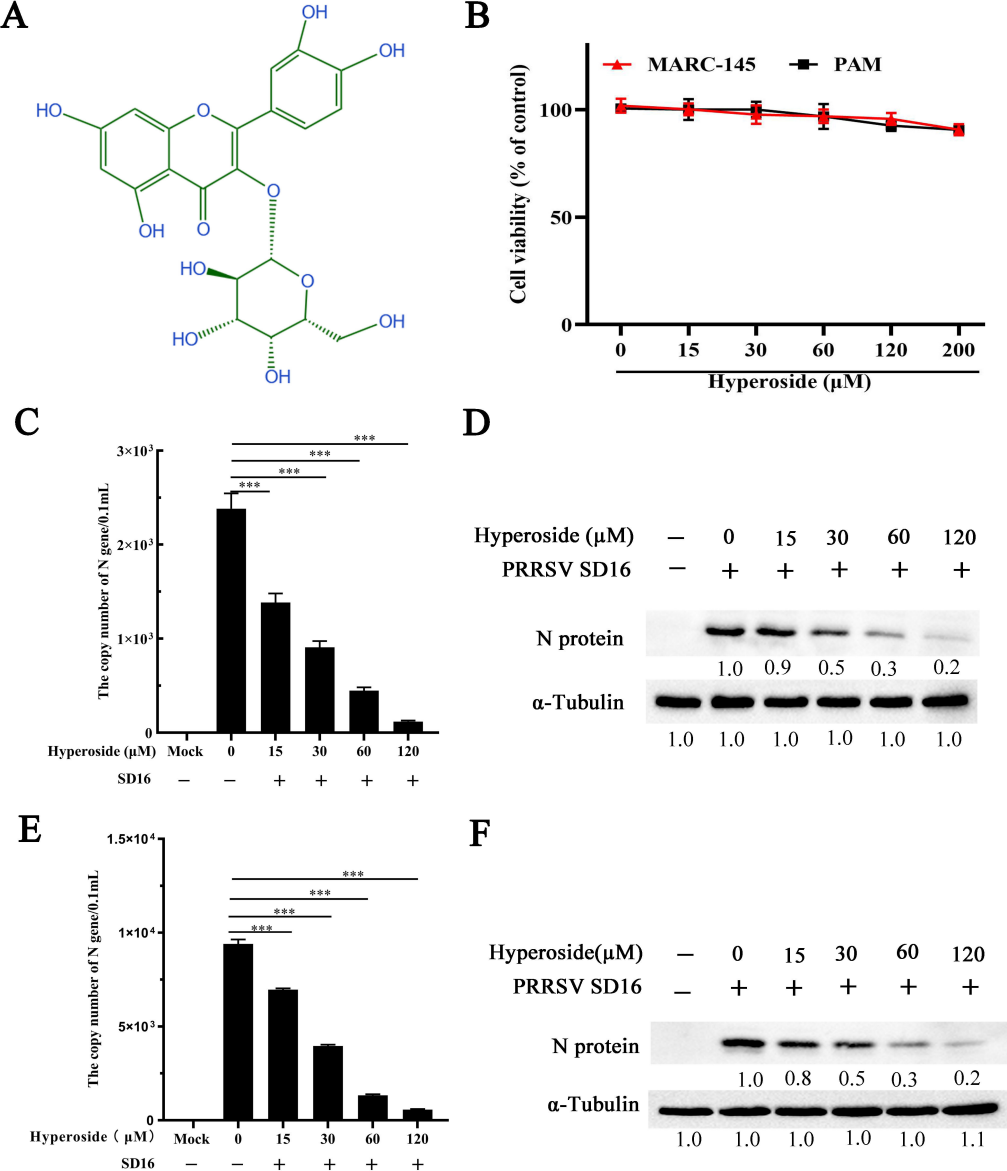
Hyperoside suppresses PRRSV proliferation in MARC-145 and PAMs. (**A**) Chemical structure of hyperoside. (**B**) The cytotoxicity of hyperoside to MARC-145 cells and PAMs. The cells were preseeded into 96-well plates and treated with hyperoside (0, 15, 30, 60, 120, or 200 µM) for 24 h, and the cell viability was measured via a CCK-8 kit. MARC-145 cells were incubated with PRRSV SD16 (MOI = 0.1) after pretreatment with hyperoside (15, 30, 60, or 120 µM) or DMSO (0 µM hyperoside). Progeny virus production and PRRSV N protein expression were subsequently measured via qPCR (**C**) and western blot (**D**) analysis at 24 hpi. Furthermore, the anti-PRRSV activity of hyperoside was also confirmed in PAMs (**E, F**) via the same protocol. The data shown are representative of three independent experiments and were subjected to Student’s *t*-tests. ***, *P* < 0.001, compared with 0 µM hyperoside-treated cells.

The chemical structure of hyperoside is depicted in Fig. 1A. In this study, the potential cytotoxicity of hyperoside to MARC-145 and PAM cells was detected via the CCK-8 assay. As shown in Fig. 1B, at concentrations up to 120 µM, hyperoside did not reduce cell viability.

MARC-145 and PAM cells were subsequently preincubated with different concentrations of hyperoside and infected with PRRSV SD16. These cellular supernatants and cells were collected to evaluate PRRSV replication at 24 hpi. The results revealed that hyperoside clearly decreased progeny virus production and PRRSV N protein expression in MARC-145 (Fig. 1C and D) and PAMs (Fig. 1E and F) (***, *P* < 0.001). These data indicate that hyperoside effectively inhibits PRRSV infection *in vitro*.

### Hyperoside exhibits broad-spectrum antiviral activity against different PRRSV strains

**Fig 2:**
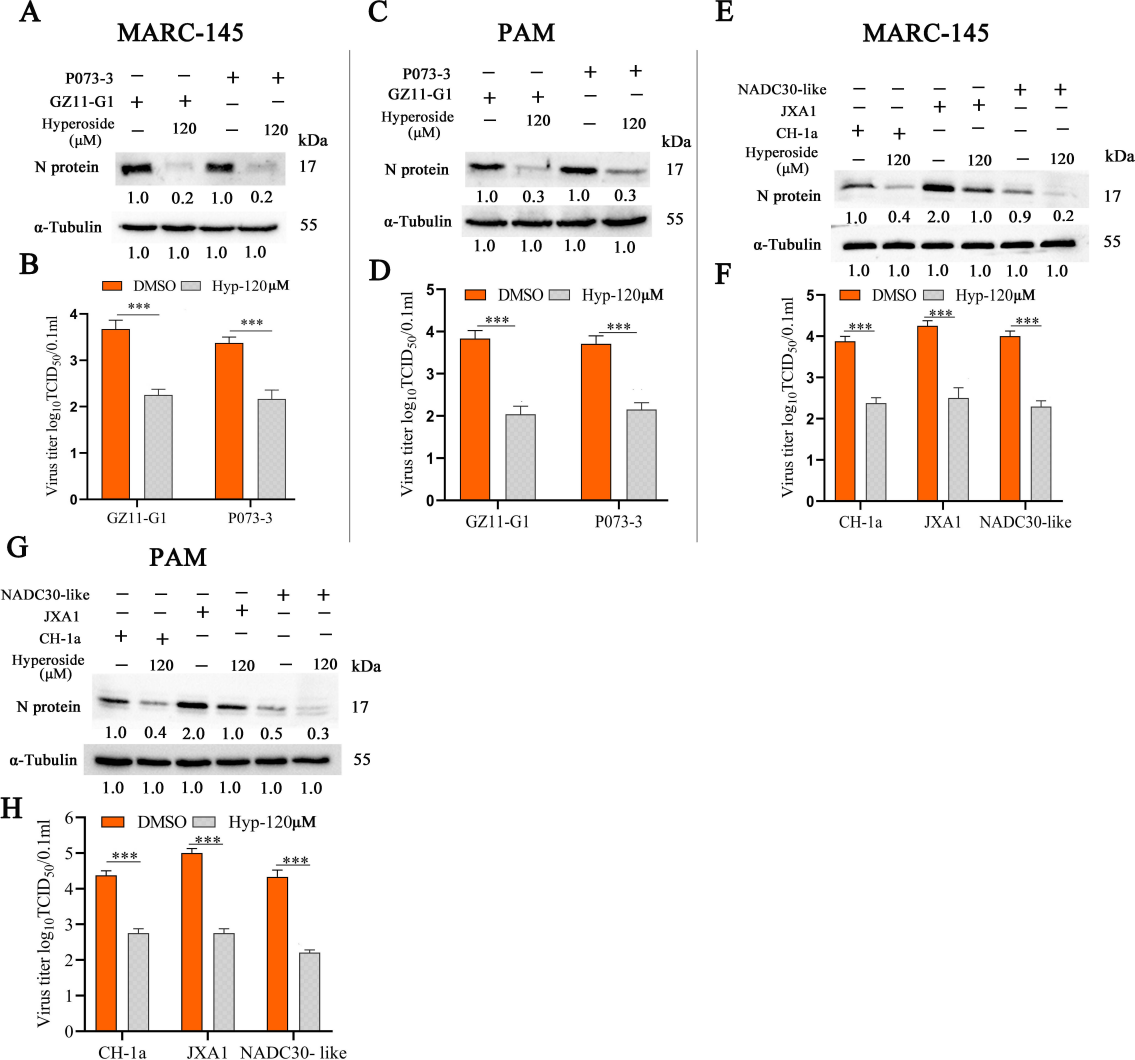
Broad-spectrum anti-PRRSV assessment of hyperoside. MARC-145 cells were incubated with or without hyperoside (120 µM) for 2 h and then infected with various PRRSV-1 strains (GZ11-G1 and P073-3) at 37°C for 1 h. The cells and cellular supernatants were harvested at 24 hpi to evaluate PRRSV N protein expression via western blot analysis (**A**), and the progeny virus titer was determined via TCID_50_ analysis (**B**). Moreover, the antiviral effects of hyperoside against PRRSV GZ11-G1 and P073-3 were also evaluated in PAMs. These cells were collected at 24 hpi to monitor N protein expression via western blot analysis (**C**). The cellular supernatants were also harvested to detect progeny virus production on the basis of the TCID_50_ value (**D**). MARC-145 cells were pretreated with hyperoside (120 µM) or DMSO for 1 h and then incubated with different PRRSV-2 strains (CH-1a, JXA1, and NADC30-like; MOI 0.1) for 1 h. All cells and cellular supernatants were collected at 24 hpi to assess PRRSV N protein expression (**E**) and progeny virus titer (**F**). The antiviral effects of hyperoside against the PRRSV-2 strains CH-1a, JXA1, and NADC30-like were subsequently assessed in PAMs. PRRSV N protein expression was monitored at 24 hpi via western blot analysis (**G**), and the progeny virus titer was examined on the basis of the TCID_50_ value (**H**). GAPDH served as the reference gene, and α-Tubulin served as the loading control. All the data are expressed as the means ± SDs and were subjected to Student’s *t*-tests. ***, *P* < 0.001, compared with DMSO-treated cells challenged with the same virus.

To further determine whether hyperoside has antiviral effects on different PRRSV-1 and PRRSV-2 strains, MARC-145 and PAM cells were preincubated with hyperoside (120 µM) and infected with various PRRSV-1 strains (GZ11-G1 or P073-3). The cells and cellular supernatants were collected at 24 hpi to assess PRRSV replication via western blot and TCID_50_ analyses. The results revealed that N protein expression was significantly lower in the hyperoside treatment group than in the DMSO treatment group (Fig. 2A). Moreover, progeny virus production was significantly lower in the hyperoside treatment group than in the DMSO treatment group (Fig. 2B) (***, *P* < 0.001). Notably, hyperoside had similar antiviral effects against the GZ11-G1 and P073-3 strains of PRRSV-1 in PAMs (Fig. 2C and D) (***, *P* < 0.001). MARC-145 and PAMs were subsequently pretreated with hyperoside (120 µM) and infected with different PRRSV-2 strains (CH-1a, JXA1, and NADC30-like). Western blot and TCID_50_ assays were employed to assess PRRSV replication at 24 hpi. Hyperoside significantly decreased PRRSV N protein expression and progeny virus production in both MARC-145 cells (Fig. 2E and F) and PAMs (Fig. 2G and H) (***, *P* < 0.001). These results showed that hyperoside exerts broad-spectrum antiviral effects on different PRRSV-1 and PRRSV-2 strains.

### Hyperoside inhibits PRRSV infection at multiple stages

**Fig 3:**
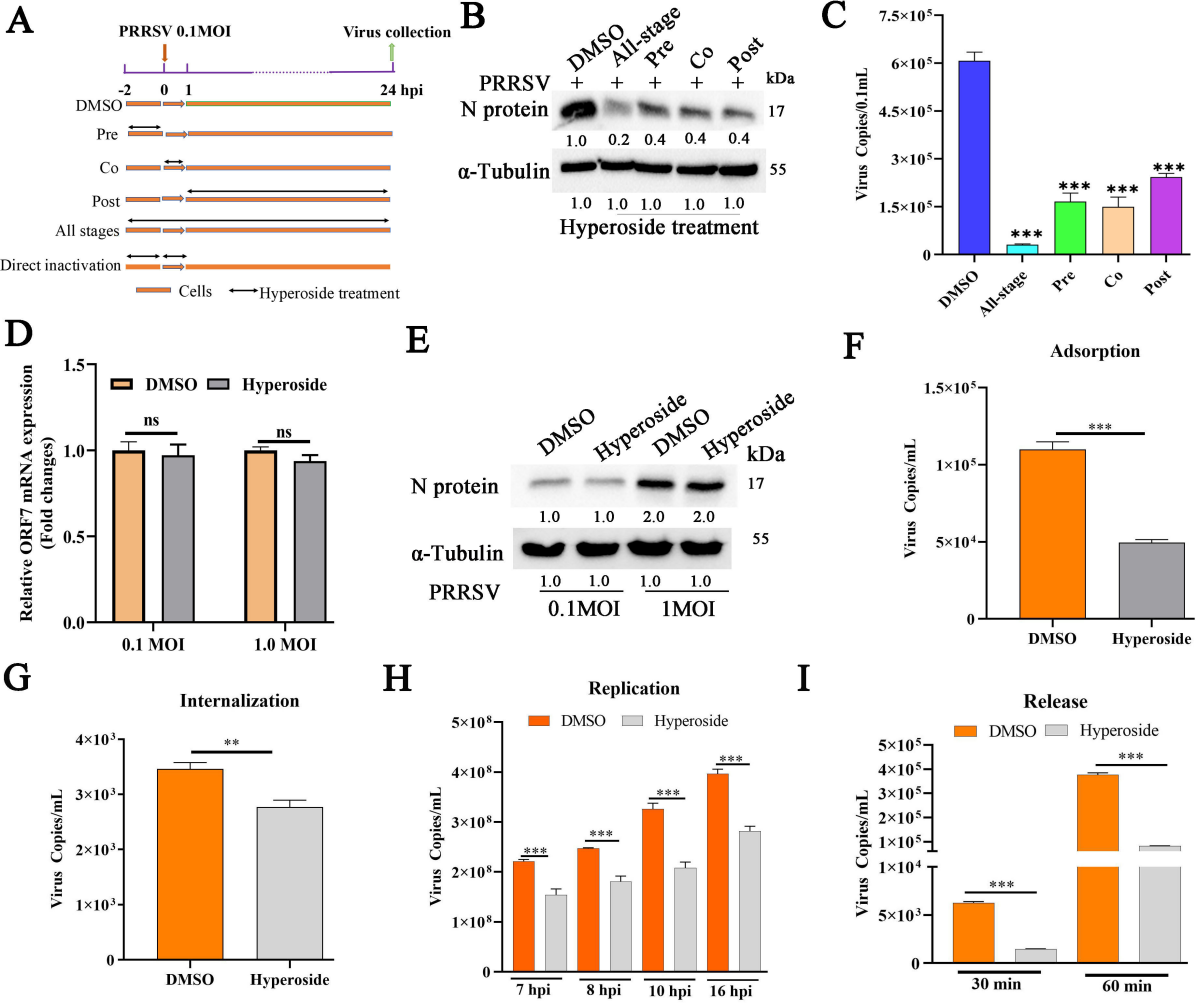
Hyperoside inhibits PRRSV infection at multiple stages. (**A**) Schematic illustration of the time-of-addition experiment. MARC-145 cells were infected with PRRSV SD16 (MOI = 0.1) and treated with hyperoside (120 µM) at different time points relative to infection (pretreatment, cotreatment, post-treatment, and continuous treatment). These cells and cellular supernatants were harvested to evaluate viral replication via western blotting (**B**) and qPCR (**C**) at 24 hpi. For the direct inactivation experiment, PRRSV (MOI = 0.1 and 1) and hyperoside (120 µM) were mixed and incubated at 37°C for 2 h. Subsequently, the mixtures were incubated with MARC-145 cells at 37°C for 1 h. These cells were collected at 24 hpi to test ORF7 expression at the mRNA (**D**) and protein levels (**E**). GAPDH was used as the reference gene, while α-Tubulin was used as the loading control. ns: not significant, compared with the DMSO-treated group. (**F**) Results of the adsorption assay. MARC-145 cells were incubated with a mixture of hyperoside or DMSO and PRRSV SD16 (MOI = 0.1) for 1 h at 4°C, after which the cells were washed and incubated at 37°C for 24 h. The number of progeny virus copies was finally tested via qPCR. (**G**) Results of the internalization assay. MARC-145 cells were pretreated with hyperoside (120 µM) for 12 h and then incubated with PRRSV SD16 (MOI = 0.1) at 4°C for 1 h. The cells were subsequently washed and finally incubated with 120 µM hyperoside or DMSO for another 1 h at 37°C. The number of progeny virus copies was ultimately tested via qPCR at 24 hpi. (**H**) Results of the replication assay. MARC-145 cells were infected with PRRSV SD16 (MOI = 1) for 6 h, and the culture medium was replaced with 3% FBS DMEM containing hyperoside (120 µM) or DMSO. The cells were collected at 7, 8, 10, and 16 hpi to detect PRRSV genome copies via qPCR. (**I**) Results of the release assay. MARC-145 cells were infected with PRRSV SD16 (MOI = 1) for 24 h, washed with PBS, and then incubated with 3% FBS DMEM containing hyperoside (120 µM) or DMSO. The cell supernatants were harvested to quantify the number of released virus particles at 30 and 60 min via qPCR. All the assays were repeated three times. The error bars represent the means ± SDs. **, *P* < 0.01; ***, *P* < 0.001, compared with the DMSO-treated group.

To investigate which stage of the PRRSV life cycle is affected by hyperoside, a time-of-addition assay was performed, as illustrated in Fig. 3A. MARC-145 cells were pretreated, cotreated, or post-treated with hyperoside prior to PRRSV inoculation. These cells and cellular supernatants were collected at 24 hpi to analyze PRRSV N protein expression and progeny virus production. As shown in Fig. 3B, N protein expression was significantly lower in the hyperoside pretreated, cotreated, and post-treated groups than in the PRRSV infection-only group. Notably, a lower viral load was observed in all stages (Fig. 3C) (***, *P* < 0.001).

To further determine whether hyperoside can directly inactivate PRRSV, hyperoside was mixed with different loads of PRRSV SD16 (MOI = 0.1 or 1) and preincubated at 37°C for 2 h. Then, MARC-145 cells were infected with PRRSV SD16, and qPCR and western blotting were used to evaluate PRRSV replication. As shown in Fig. 3D and E, the protein and mRNA expression levels of PRRSV N did not significantly differ between the hyperoside-treated group and the DMSO-treated group.

Previous studies have reported that the infectious cycle of PRRSV involves various stages, such as adsorption, entry, replication, and release (42, 43). Therefore, we subsequently performed a viral adsorption assay, a viral entry assay, a viral replication assay, and a viral release assay to determine the specific phase during which hyperoside exerts anti-PRRSV effects in MARC-145 cells. As depicted in Fig. 3F, compared with DMSO treatment, hyperoside treatment obviously decreased the copy number of PRRSV progeny viruses in MARC-145 cells during the adsorption stage. Similar anti-PRRSV effects were also observed in MARC-145 cells during entry (Fig. 3G), replication (Fig. 3H), and release (Fig. 3I) (**, *P* < 0.01; ***, *P* < 0.001). These data suggest that hyperoside can suppress PRRSV infection at multiple stages, although it does not exert any direct virucidal effect on PRRSV.

### Hyperoside significantly attenuates PRRSV-induced pro-inflammatory cytokine production in susceptible cells

**Fig 4:**
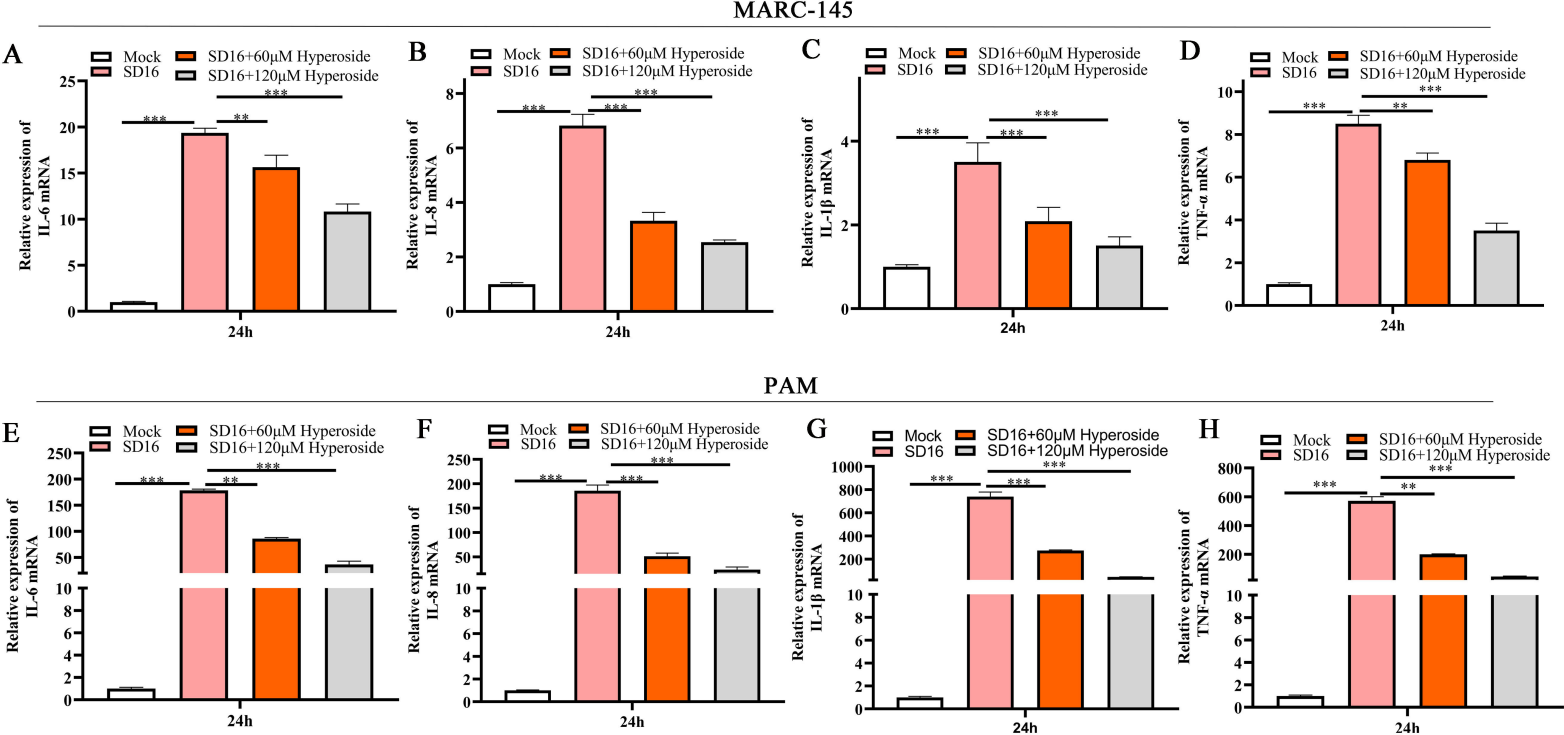
Hyperoside alleviates the pro-inflammatory response in infected MARC-145 cells and PAMs. MARC-145 cells were seeded into 12-well plates and infected with PRRSV SD16 (MOI = 0.1) in the presence of hyperoside (120 µM) or 0.1% DMSO. These cells were harvested at 24 hpi to detect the expression of pro-inflammatory cytokines, including IL-6 (**A**), IL-8 (**B**), IL-1β (**C**), and TNF-α (**D**), via qPCR. The experiment was repeated in PAMs, and the relative expression of IL-6 (**E**), IL-8 (**F**), IL-1β (**G**), and TNF-α (**H**) in PAMs was assessed via qPCR. GAPDH served as the internal control. **, *P* < 0.01; ***, *P* < 0.001, compared with the SD16-infected group.

Numerous studies have shown that PRRSV infection induces the release of pro-inflammatory factors, promoting pathogenicity and lung damage (22, 44). Moreover, evidence from recent research suggests that hyperoside can exert potent anti-inflammatory effects under some pathogenic conditions by regulating inflammatory cytokine production (34, 45). To test whether hyperoside modulates the expression of pro-inflammatory factors induced by PRRSV infection, MARC-145 cells and PAMs were treated with hyperoside (60 or 120 µM) and then infected with PRRSV SD16. The cells were collected at 24 hpi to analyze IL-6, IL-8, IL-1β, and TNF-α expression. As depicted in Fig. 4A through D, hyperoside significantly reduced the IL-6, IL-8, IL-1β, and TNF-α levels in MARC-145 cells (**, *P* < 0.01; ***, *P* < 0.001). Similar results were also observed in PAMs (Fig. 4E through H). Consistent with the mRNA levels, hyperoside significantly reduced the production of IL-6, IL-8, IL-1β, and TNF-α in the supernatants of PRRSV-infected PAMs (Fig. S1) (**, *P* < 0.01; ***, *P* < 0.001). These findings indicate that hyperoside can attenuate the pro-inflammatory cytokine responses induced by PRRSV infection.

### Hyperoside inhibits the TLR4/NF-κB signaling pathway to suppress PRRSV infection

**Fig 5:**
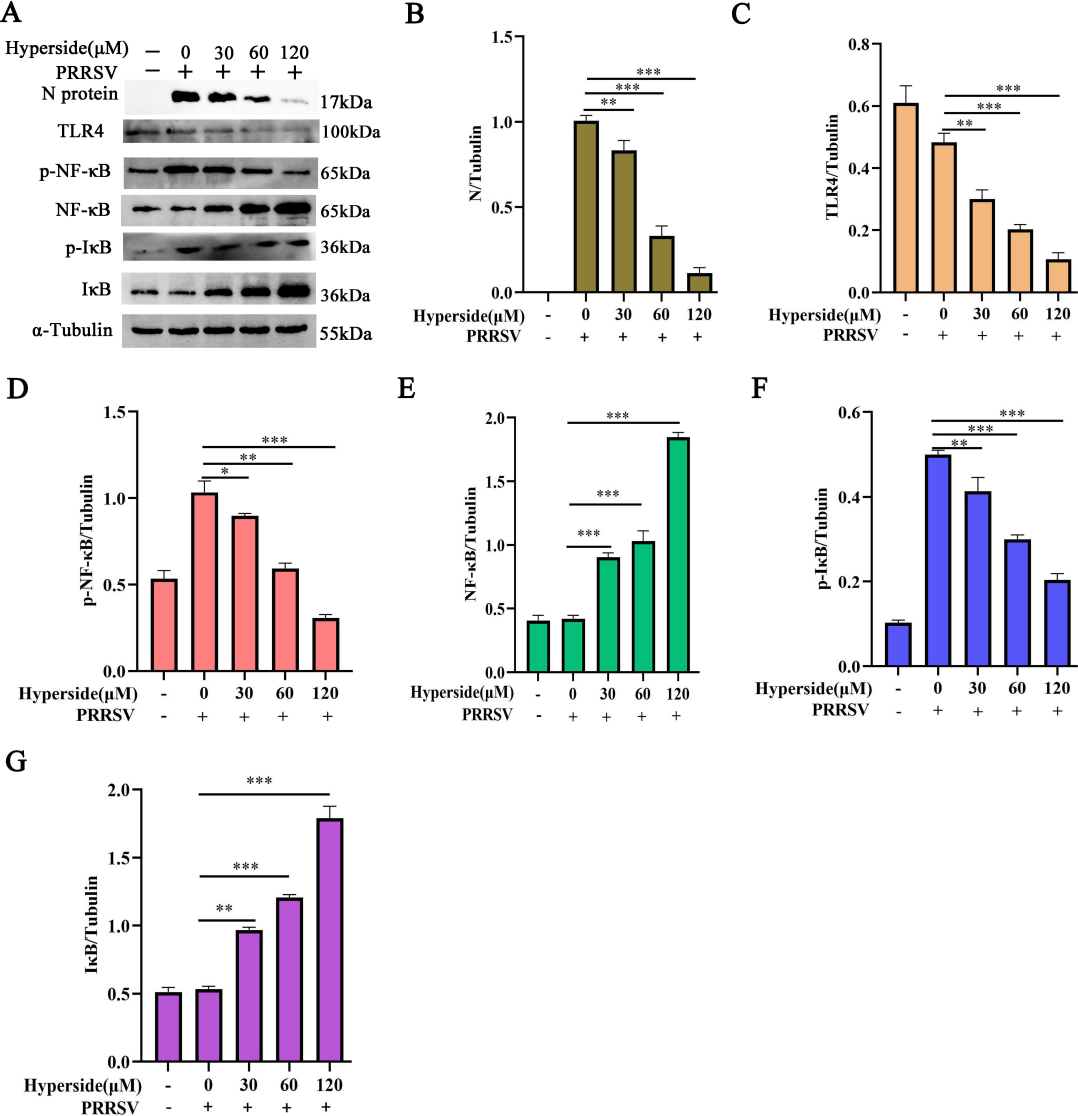
Hyperoside inhibits the inflammation induced by PRRSV by regulating the TLR4/NF-κB signaling pathway. MARC-145 cells were added to 12-well plates and incubated with different concentrations of hyperoside (30, 60, and 120 µM) for 2 h. These cells were then infected with PRRSV SD16 (MOI = 1) for 1 h. At 24 hpi, the cells were collected, and N protein, TLR4, p-NF-κB p65, NF-κB p65, p-IκBα, and IκBα protein expression was detected via western blotting (**A**). The quantitative analysis of the PRRSV N protein (**B**), TLR4 (**C**), p-NF-κB p65 (**D**), NF-κB p65 (**E**), p-IκBα (**F**), and IκBα (**G**) levels was conducted via ImageJ. The values are presented as the means ± SDs. *, *P* < 0.05; **, *P* < 0.01; ***, *P* < 0.001, compared with only PRRSV-infected cells.

The TLR4/NF-κB signaling pathway plays important roles in pro-inflammatory responses, cell differentiation, proliferation, and apoptosis (46). More specifically, TLR4, which is expressed on the cell surface, serves as an innate immune receptor and plays a pivotal role in initiating innate immune responses (47). NF-κB, which is downstream of TLR4, is a key transcription factor involved in inflammatory responses in mammalian cells (48). To explore the mechanisms underlying the anti-inflammatory effects of hyperoside during PRRSV infection, TLR4/NF-κB signaling pathway-related protein expression was detected via western blotting. As depicted, the levels of phosphorylated NF-κB p65 (p-NF-κB p65) and phosphorylated IκB (p-IκB) in MARC-145 cells were significantly greater in the infection group than in the mock group (Fig. 5). Hyperoside treatment at the indicated concentrations decreased the expression of TLR4 and the levels of p-NF-κB p65 and p-IκB both in the presence (Fig. 5) and absence (Fig. S2) of PRRSV infection (**, *P* < 0.01; ***, *P* < 0.001). However, the levels of NF-κB p65 and IκB were significantly increased by hyperoside treatment in a dose-dependent manner (Fig. S2). Thus, TLR4 is the primary receptor that mediates the anti-inflammatory effects of hyperoside. siRNA-mediated knockdown of TLR4 in MARC-145 or PAM cells reversed the inhibitory effects of hyperoside on pro-inflammatory factors (Fig. S3) (***, *P* < 0.001). These data indicate that hyperoside treatment attenuates the expression of pro-inflammatory factors induced by PRRSV via the inhibition of the TLR4/NF-κB signaling pathway.

### Hyperoside reduces PRRSV-induced autophagy

**Fig 6:**
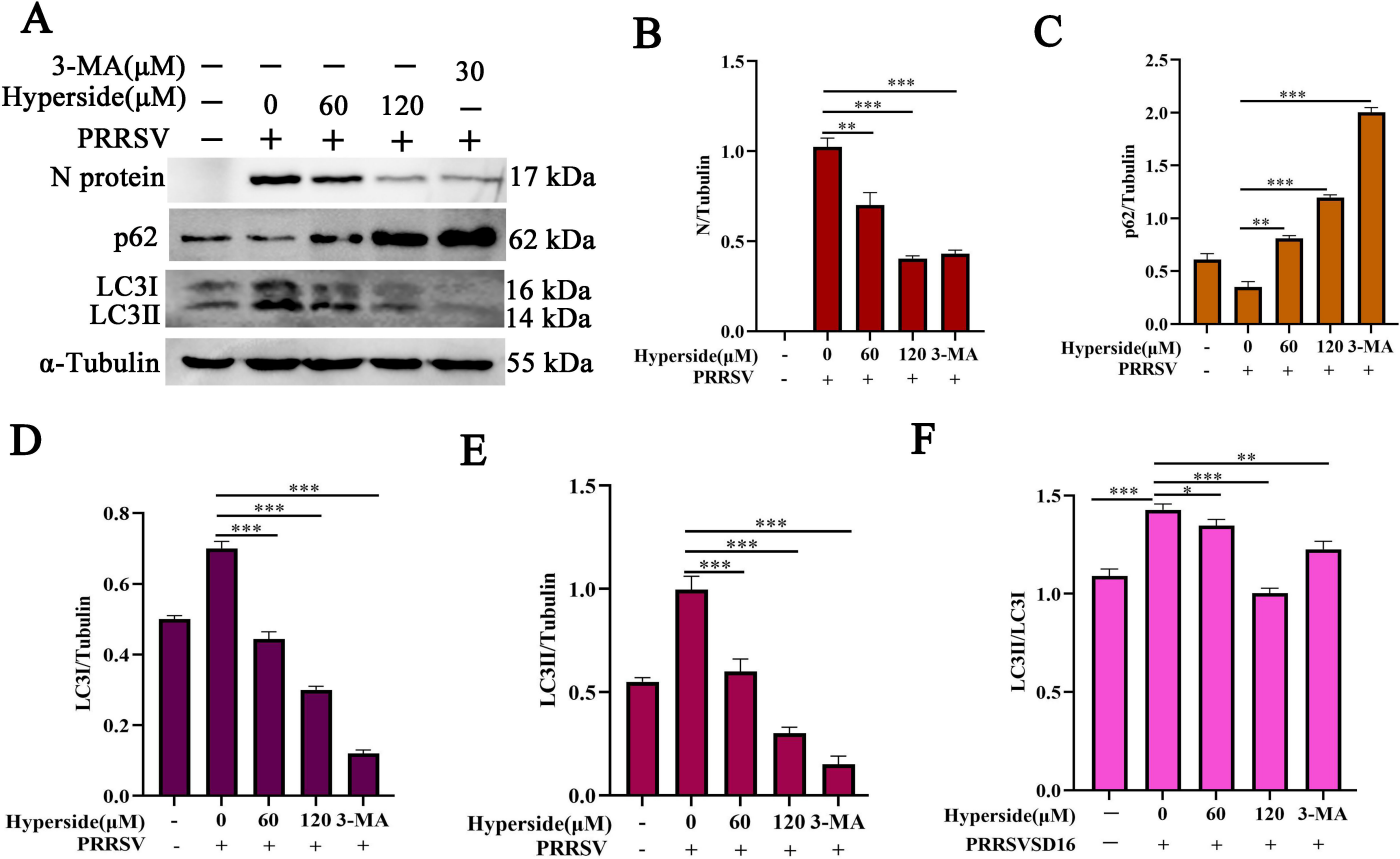
Hyperoside inhibits PRRSV-induced autophagy. MARC-145 cells were pretreated with hyperoside (60 and 120 µM) or 3-MA (30 µM) for 2 h and then infected with PRRSV SD16 (MOI = 0.5) for 1 h, after which the medium was subsequently replaced with 3% FBS DMEM containing the indicated concentrations of hyperoside or 3-MA. After 24 h, the cells were harvested to analyze the expression of autophagy-related proteins (p62 and LC3I or LC3II) and the PRRSV N protein via western blotting (**A**). The quantitative analysis of the levels of the PRRSV N protein (**B**), p62 (**C**), LC3I (**D**), and LC3II (**E**), and the LC3II/LC3I ratio (**F**) was conducted via ImageJ. The values are presented as the means ± SDs. *, *P* < 0.05; **, *P* < 0.01; ***, *P* < 0.001, compared with only PRRSV-infected cells.

The autophagy induced by viral infection can promote viral replication. Previous studies have shown that PRRSV can hijack the autophagy pathway to increase viral self-replication (29, 49). Interestingly, hyperoside has been found to attenuate rotenone-induced neuronal injury and sepsis-induced acute lung injury via the regulation of autophagy (33, 34). To test whether hyperoside inhibits PRRSV infection via the suppression of autophagy, MARC-145 cells were pretreated with different concentrations of hyperoside (60 or 120 µM) or 3-AM (30 µM) and infected with PRRSV SD16 (MOI = 0.5). As depicted in Fig. 6, compared with mock-infected MARC-145 cells, PRRSV-infected MARC-145 cells presented an obvious band pattern transformation from LC3I to LC3II, and the ratio of LC3II/LC3I was increased, leading to degradation of p62. Moreover, hyperoside significantly inhibited PRRSV-induced autophagy in a dose-dependent manner. Notably, both hyperoside and 3-MA significantly decreased PRRSV N protein expression (*, *P* < 0.05; **, *P* < 0.01; ***, *P* < 0.001).

**Fig 7:**
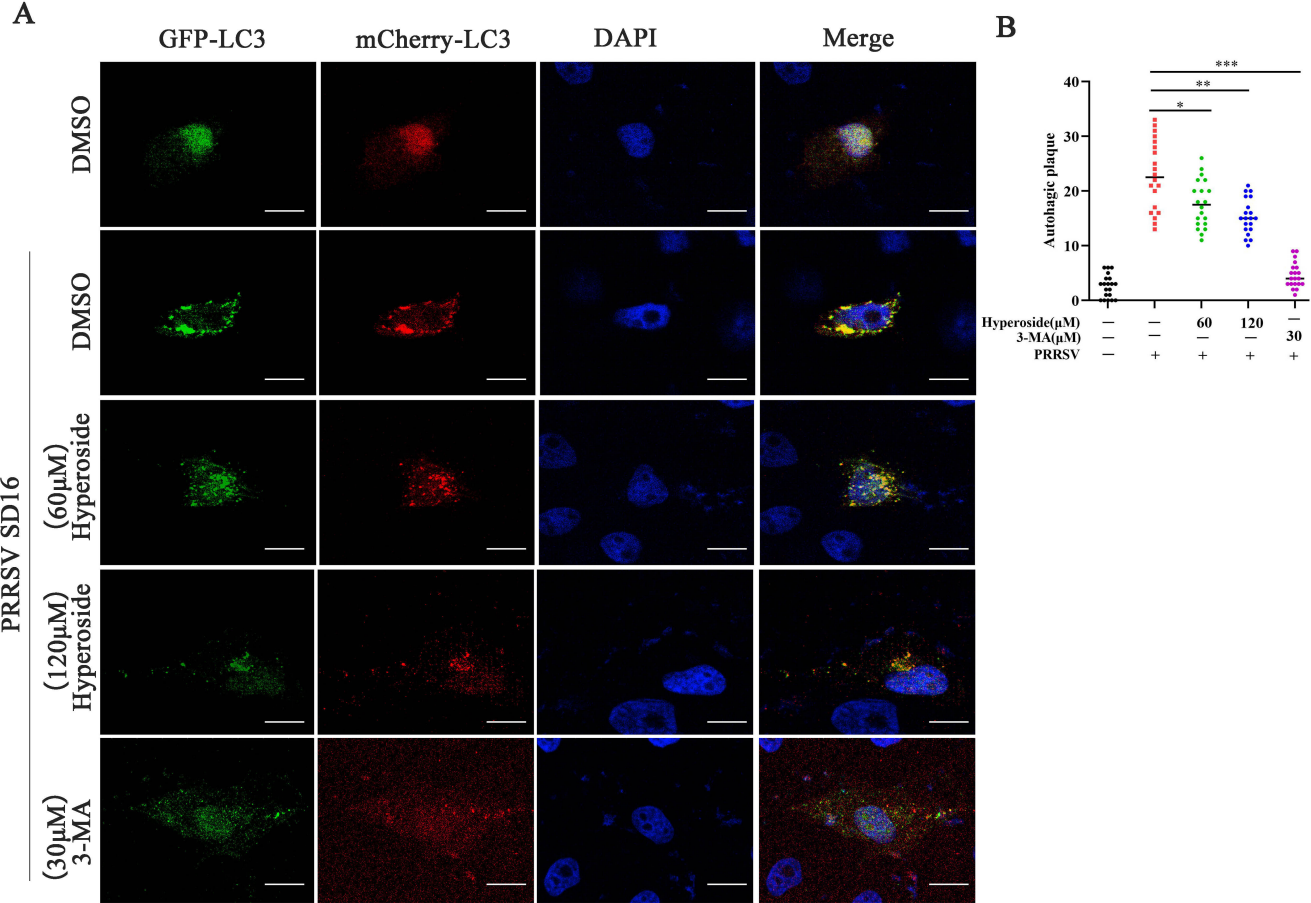
Hyperoside decreases PRRSV replication by alleviating autophagy. MARC-145 cells were transfected with p-mCherry-GFP-LC3 for 12 h and treated with hyperoside (60 or 120 µM) or 3-MA (30 µM) for 2 h. These cells were then infected with PRRSV SD16 (MOI = 0.5) for 1 h, and the medium was subsequently replaced with 3% FBS DMEM containing the indicated concentrations of hyperoside or 3-MA. After 24 h, the cells were fixed, and their nuclei were stained with DAPI (blue). The LC3 puncta were visualized via confocal microscopy (**A**). Scale bar = 10 µm. LC3 puncta formation was quantified in MARC-145 cells (**B**). The results represent the number of LC3 puncta per cell in panel A (*n* = 20).

Fluorescence microscopy was performed to confirm the effect of hyperoside on PRRSV-induced autophagy. The results revealed that PRRSV infection promoted autophagic plaque formation, but these changes were reversed by hyperoside in PRRSV-infected MARC-145 cells (Fig. 7) (*, *P* < 0.05; **, *P* < 0.01; ***, *P* < 0.001). These findings demonstrated that hyperoside can inhibit PRRSV replication by attenuating virus-induced autophagy.

### Hyperoside attenuates PRRSV-induced autophagy through p62/Nrf2/Keap1 signaling axis activation

**Fig 8:**
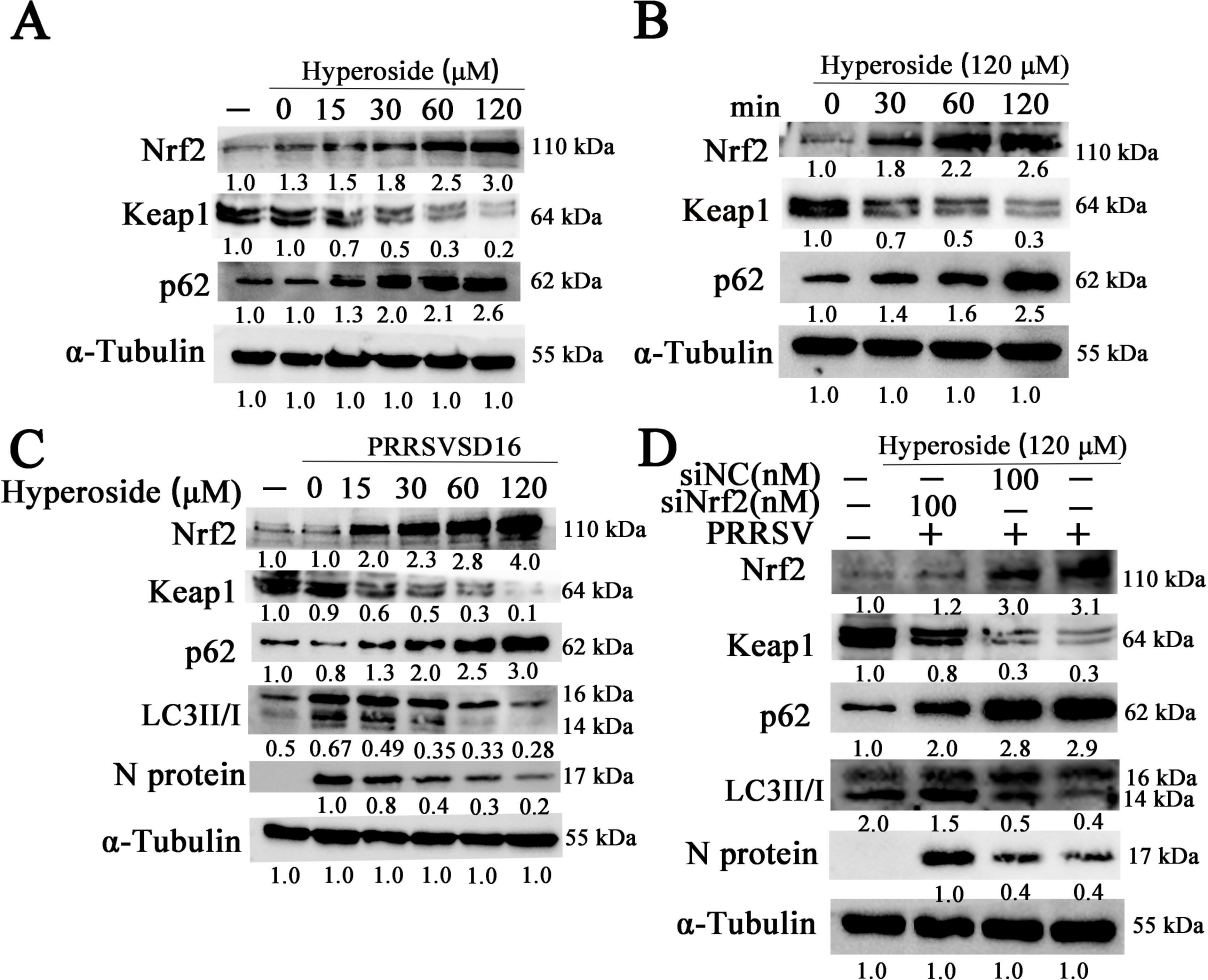
Hyperoside decreases PRRSV-induced autophagy via p62/Nrf2/Keap1 signal axis activation. (**A**) MARC-145 cells were seeded into 12-well plates and incubated with different concentrations of hyperoside (0, 15, 30, 60, and 120 µM) for 24 h. These cells were collected to analyze Nrf2, Keap1, and p62 expression via western blot analysis. (**B**) MARC-145 cells were pretreated with hyperoside (120 µM) and collected at 30, 60, and 120 min, and Nrf2, Keap1, and p62 expression were examined via western blot analysis. (**C**) MARC-145 cells were pretreated with hyperoside at different dosages, infected with 0.5 MOI PRRSV SD16, and then harvested to detect Nrf2, Keap1, p62, LC3I, LC3II, and N protein expression at 24 hpi via western blot analysis. (**D**) MARC-145 cells were transfected with siNrf2 or siNC for 12 h and then incubated with hyperoside (120 µM) for 2 h. Subsequently, the cells were infected with PRRSV SD16 (0.5 MOI). These cells were harvested at 24 hpi to detect Nrf2, Keap1, p62, LC3I, LC3II, and N protein expression via western blot analysis. α-Tubulin served as the internal control.

The p62/Nrf2/Keap1 signaling pathway has been implicated in numerous cellular functions, such as autophagy, apoptosis, and ferroptosis (50–53). In this study, we first tested whether hyperoside could promote p62/Nrf2/Keap1 activation. MARC-145 cells were treated with different concentrations of hyperoside for 24 h, and the total cell lysates were harvested and analyzed via western blotting. As shown in Fig. 8A, hyperoside increased the accumulation of p62 and total Nrf2 in MARC-145 cells in a dose-dependent manner. Moreover, it induced a corresponding decrease in Keap1 expression. Moreover, p62, Nrf2, and Keap1 expression in MARC-145 cells incubated with hyperoside were also detected at different time points via western blotting. As shown in Fig. 8B, hyperoside increased the accumulation of p62 and total Nrf2 in MARC-145 cells in a time-dependent manner, and Keap1 expression also decreased after 2 h of incubation with hyperoside. A previous study reported that hyperoside decreases rotenone-induced neuronal injury via the inhibition of autophagy (33). To further test whether the anti-PRRSV activity of hyperoside is related to autophagy regulation by the p62/Nrf2/Keap1 axis activation, MARC-145 cells were treated with different concentrations of hyperoside and infected with PRRSV SD16. These cells were collected at 24 hpi to analyze p62, Nrf2, Keap1, LC3II/I, and PRRSV N protein expression via western blot analysis. As shown in Fig. 8C, hyperoside decreased the ratio of LC3II/I and downregulated N protein expression in MARC-145 cells while increasing p62 and Nrf2 expression. In addition, we found that p62 and Nr2 were colocalized in the nucleus in PRRSV-infected cells treated with hyperoside (Fig. S4).

These data suggest that hyperoside may promote the nuclear translocation of Nrf2 to increase the transcription and expression of p62.

In another set of experiments, MARC-145 cells were transfected with siNrf2 or siNC, treated with hyperoside, and subsequently infected with PRRSV. Nrf2, Keap1, LC3I, LC3II, p62, and N protein expression in these cells were subsequently analyzed. As expected, siNrf2 attenuated the effects of hyperoside on the LC3II/I ratio and N protein expression (Fig. 8D). Collectively, these findings demonstrated that hyperoside exerts anti-PRRSV effects by suppressing PRRSV-induced autophagy via the p62/Nrf2/Keap1 signaling pathway.

### Hyperosides exhibit anti-PRRSV activity in piglets

**Fig 9:**
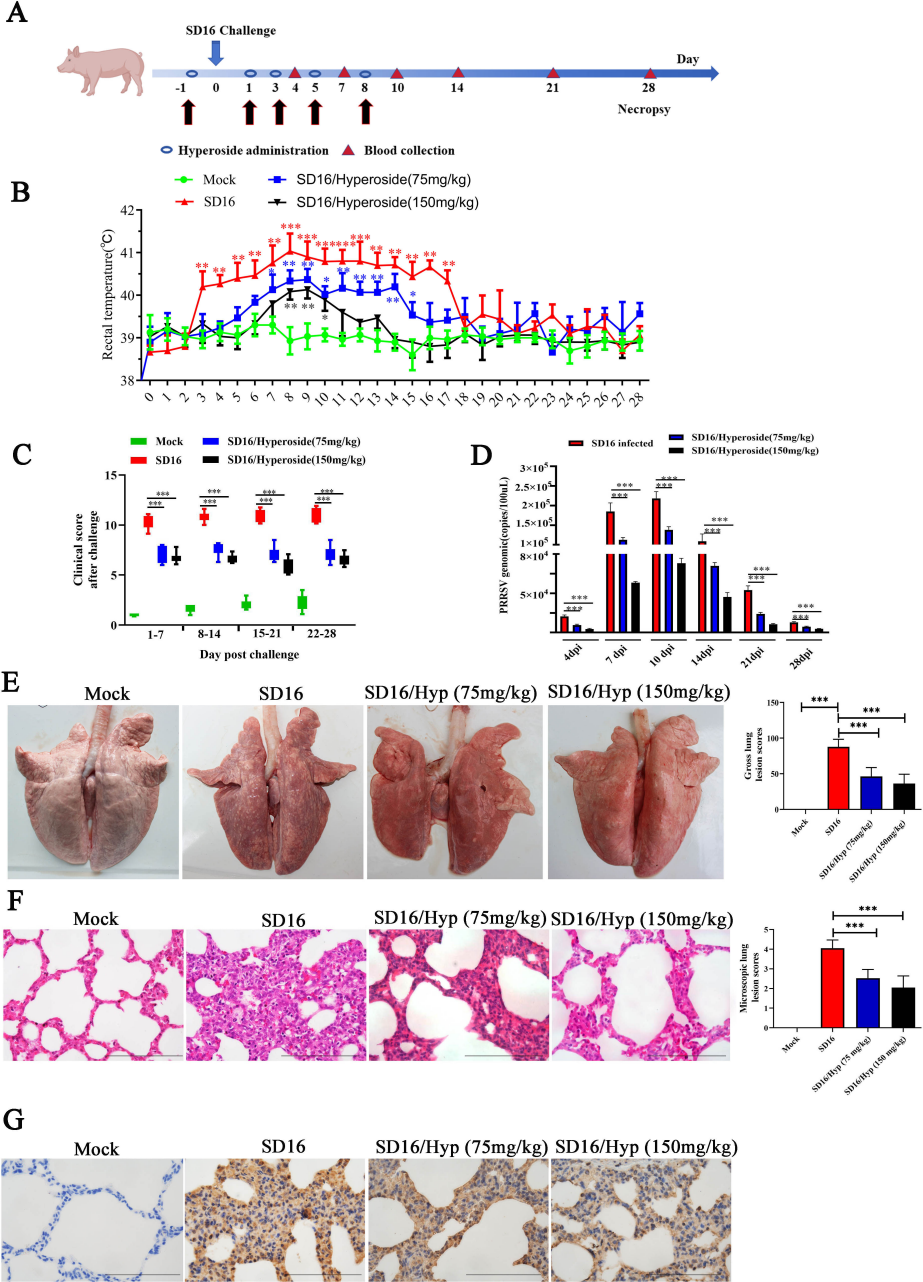
Hyperoside protects against PRRSV infection in piglets. (**A**) Schematic diagram of the PRRSV infection protocol in piglets. Piglets were orally pretreated with 75 or 150 mg/kg hyperoside or DMSO on day −1 and then intranasally inoculated with PRRSV SD16 (3 × 10^5^ TCID_50_/piglet) or MEM. Hyperoside was readministered at the pretreatment dose at the indicated time point after viral infection. (**B**) The rectal temperature (°C) of all piglets was measured daily after the PRRSV challenge. The data are presented as the means ± SDs. *, *P* < 0.05; **, *P* < 0.01; ***, *P* < 0.001, compared with the Mock group. (**C**) The clinical symptoms of piglets from all groups were observed. The clinical score of each group was reported as the average of the time intervals (1–7, 8–14, 15–21, 22–28 DPC). The daily total clinical score was the sum of the scores given for each group. ***P < 0.001 compared with the SD16-treated pigs. (**D**) The number of PRRSV genome copies in the serum samples of each group was determined at the indicated time points via qPCR analysis. The results are presented as the means ± SDs. ***P < 0.001. All the piglets were euthanized at 28 DPC, and gross lung lesions (**E**) and microscopic lung lesions (**F**) were observed and scored. Histograms of gross lung lesions represent the percentage of affected lung tissue. Histograms of microscopic lung lesions represent the scores of interstitial pneumonia based on five fields/slides. ***P < 0.001, compared with the SD16-infected group. Scale bars = 100 µm. (**G**) Immunohistochemical staining of lung tissues derived from piglets infected with PRRSV SD16 and treated with hyperoside (75 or 150 mg/kg) at 28 DPC via the anti-PRRSV antibody. Scale bars = 100 µm.

To further analyze the anti-PRRSV activity of hyperoside *in vivo*, piglets were challenged with PRRSV SD16 and treated with hyperoside (Fig. 9A). The rectal temperatures of all piglets were monitored daily after the PRRSV challenge. As shown in Fig. 9B, the average temperatures of the piglets in the PRRSV SD16 challenge-only group began to rise at 3 DPC, peaking at approximately 41°C by 8 DPC and remaining higher than 40°C until 17 DPC. In contrast, the rectal temperatures of the 75 mg/kg hyperoside-treated group rose slowly from 5 DPC, peaking at approximately 40.3°C by 14 DPC. Moreover, the rectal temperature of the 150 mg/kg hyperoside-treated group rose slowly from 7 DPC to a peak temperature of approximately 40.1°C at 10 DPC. In this group, high temperatures above 40°C were detected at 8–10 DPC (*, *P* < 0.05; **, *P* < 0.01; ***, *P* < 0.001). All the piglets exhibited various clinical signs of viral infection, including lethargy, a rough coat, poor appetite, dyspnea, and periocular edema. The clinical scores of piglets treated with 75 and 150 mg/kg hyperoside were significantly lower than those of untreated piglets challenged with PRRSV SD16 at 1–7, 8–14, 15–21, and 22–28 DPC (Fig. 9C) (***, *P* < 0.001). Consistent with the clinical symptom scores, the serum copy numbers of PRRSV in piglets treated with 75 or 150 mg/kg hyperoside were also obviously lower than those in untreated piglets challenged with PRRSV SD16 (Fig. 9D) (***, *P* < 0.001).

The therapeutic action of hyperoside against PRRSV-induced lung damage was also examined. We observed gross lung lesions in the piglets, and untreated piglets infected with SD16 presented more severe pulmonary consolidation than those treated with hyperoside. In contrast, no pathological lesions were identified in the negative control group (Fig. 9E) (***, *P* < 0.001). Microscopic lung lesion analysis revealed severely thickened alveolar septa and high infiltration of inflammatory cells in the SD16-only group, but these pathologies were milder in the piglets treated with 75 or 150 mg/kg hyperoside (Fig. 9F) (***, *P* < 0.001). Moreover, more PRRSV proteins were distributed in the lung tissue of the SD16-only group than in those of the hyperoside 75 mg/kg and hyperoside 150 mg/kg groups (Fig. 9G). The mean gross lesion and microscopic lesion scores of the lungs were obviously greater in the SD16-only group than in the negative control group and significantly greater than those in the hyperoside 75 mg/kg and hyperoside 150 mg/kg groups (Fig. 9E and F). However, 150 mg/kg hyperoside was more effective than 75 mg/kg hyperoside in reducing the clinical score.

## DISCUSSION

**Fig 10:**
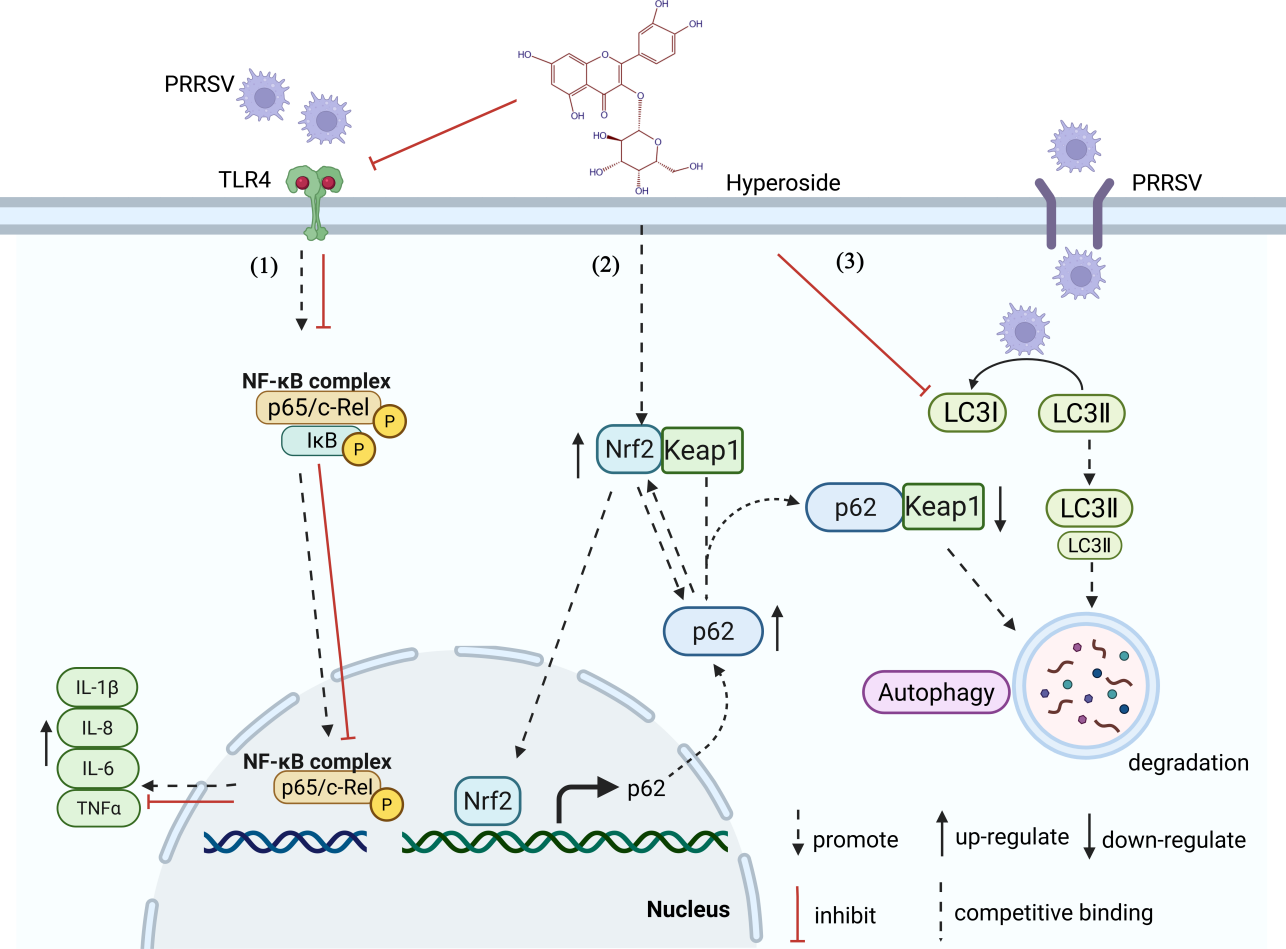
Schematic showing the mechanisms underlying the activity of hyperoside against PRRSV replication. This figure illustrates the molecular mechanisms by which hyperoside modulates the cellular response to PRRSV infection. The diagram shows three major pathways affected by hyperoside: (1) TLR4-NF-κB pathway inhibition, where PRRSV activates TLR4 receptors, leading to NF-κB complex (p65/c-Rel) activation and translocation to the nucleus, which typically upregulates inflammatory cytokines (IL-1β, IL-8, IL-6, TNFα), but hyperoside inhibits this pathway (shown by red inhibitory lines); (2) Nrf2-Keap1 pathway modulation, where hyperoside upregulates p62 protein expression, which competes with Nrf2 for binding to Keap1, allowing Nrf2 to be released and upregulated, and free Nrf2 can translocate to the nucleus to promote p62 transcription and expression; and (3) autophagy pathway regulation, where PRRSV normally promotes autophagy through LC3I/LC3II, but hyperoside inhibits this viral modulation (shown by red inhibition line to the right), promotes the p62-Keap1 interaction, leading to eventual degradation of Keap1.

PRRSV is an important pathogen that causes serious disease in pigs and hinders the development of the global swine industry. Current vaccines and drugs do not provide adequate protection against this virus, and new antiviral therapies are urgently needed. In the present study, hyperoside was found to exert potent anti-PRRSV effects in susceptible cells (Fig. 1 and 2) and piglets (Fig. 9). Furthermore, our mechanistic evaluation revealed that hyperoside alleviates pro-inflammatory responses by inhibiting the TLR4/NF-kB signaling pathway. Additionally, it suppresses PRRSV-induced autophagy via p62/Nrf2/Keap1 axis activation (Fig. 10).

Viral infection involves multiple phases, namely, binding, internalization, replication, and release (54). Recent studies have reported that hyperoside exerts antiviral effects at different stages of viral infection. For example, Wang et al. reported that hyperoside inhibits EHV-8 infection by disrupting the adsorption and internalization phases (17). In contrast, in another study, hyperoside was found to suppress SARS-CoV-2 infection by disrupting viral entry and replication (55). Furthermore, Wang et al. reported that hyperoside inhibits PEDV infection by preventing the interaction between the N protein and p53 in the replication phase (10). Notably, the results of our study demonstrated that hyperoside prevents PRRSV infection at the binding, entry, replication, and release stages without causing direct virus inactivation (Fig. 3).

The inflammatory response represents the host’s defence against viral infections. However, uncontrolled inflammatory responses usually cause severe damage to the host (56). PRRSV infection triggers the uncontrolled production of pro-inflammatory cytokines (IL-6, IL-8, IL-1β, and TNF-α) to promote self-replication (57). Interestingly, our study revealed that hyperoside can attenuate the upregulation of IFN-β, IL-6, IL-8, and TNF-α induced by PRRSV infection in susceptible cells (Fig. 4). NF-κB is a crucial mediator of inflammatory responses (58), and previous studies have shown that HP-PRRSV can induce pro-inflammatory cytokine production by regulating the TAK-1/NF-κB signaling pathway (59). Kim et al. reported that hyperoside exerts anti-inflammatory effects by blocking NF-κB activation in mouse peritoneal macrophages (60). In the present study, we explored the anti-inflammatory mechanisms of hyperoside against PRRSV infection. Our results revealed that hyperoside treatment reduces TLR4, p-NF-κB p65, and p-IκB expression in virus-infected cells (Fig. 5). Our data suggest that hyperoside can attenuate PRRSV-induced inflammatory storms by inhibiting the TLR4/NF-κB signaling pathway.

The relationship between viral replication and autophagy has been explored extensively. Autophagy can serve as a pro- or antiviral process during virus infection (61). Diao et al. demonstrated that PRRSV infection induces autophagy via endoplasmic reticulum stress-induced calcium signaling, which promotes viral replication (40). In the present study, we demonstrated that hyperoside obviously decreased the ratio of LC3II/I expression in PRRSV-infected cells in a dose-dependent manner (Fig. 6). Additionally, we found that hyperoside can inhibit PRRSV-induced autophagic plaque formation in susceptible cells (Fig. 7). In addition, our findings revealed that hyperoside has powerful anti-PRRSV activity both *in vitro* and *in vivo*. However, the present study has several limitations, and further investigations are needed to explore the direct targets of hyperoside.

### Conclusions

In conclusion, our results revealed that hyperoside inhibited viral binding, entry, replication, and release during PRRSV infection in a dose-dependent manner. In addition, hyperoside alleviated the inflammatory responses and autophagy caused by PRRSV by suppressing the TLR4/NF-κB pathway and activating the p62/Nrf2/Keap1 axis. Our findings suggest that hyperoside has potential as a promising therapeutic candidate for PRRSV control, warranting further investigations and preclinical studies to evaluate its efficacy and safety.

## Data Availability

All the data generated or analyzed during this study are included in this published article and its additional files.

## SUPPLEMENTAL MATERIAL

Supplemental material spectrum.03107-24-s0001.docx Fig. S1 to S4.

ASM does not own the copyrights to Supplemental Material that may be linked to, or accessed through, an article. The authors have granted ASM a non-exclusive, world-wide license to publish the Supplemental Material files. Please contact the corresponding author directly for reuse.

## References

[1] Dokland T. 2010. The structural biology of PRRSV. Virus Res 154:86–97. doi:10.1016/j.virusres.2010.07.029 20692304 PMC7114433

[2] Liu B, Luo L, Shi Z, Ju H, Yu L, Li G, Cui J. 2023. Research progress of porcine reproductive and respiratory syndrome virus NSP2 protein. Viruses 15:2310. doi:10.3390/v15122310 38140551 PMC10747760

[3] Cai H, Zhang H, Cheng H, Liu M, Wen S, Ren J. 2023. Progress in PRRSV infection and adaptive immune response mechanisms. Viruses 15:1442. doi:10.3390/v15071442 37515130 PMC10385784

[4] Li Y, Wang Y, Pei X, Chen S, Jing Y, Wu Y, Ma Z, Li Z, Zheng Z, Feng Y, Xu L, Liu X, Guo X, Zheng H, Xiao S. 2024. A chimeric strain of porcine reproductive and respiratory syndrome virus 2 derived from HP-PRRSV and NADC30-like PRRSV confers cross-protection against both strains. Vet Res 55:132. doi:10.1186/s13567-024-01390-y 39375803 PMC11460240

[5] Sun Q, Xu H, An T, Cai X, Tian Z, Zhang H. 2023. Recent progress in studies of porcine reproductive and respiratory syndrome virus 1 in China. Viruses 15:1528. doi:10.3390/v15071528 37515213 PMC10384046

[6] Zhu Z, Yuan L, Hu D, Lian Z, Yao X, Liu P, Li X. 2022. Isolation and genomic characterization of a Chinese NADC34-like PRRSV isolated from Jiangsu province. Transbound Emerg Dis 69:e1015–e1027. doi:10.1111/tbed.14392 34786872

[7] Wang Q, Wei HC, Zhou SJ, Li Y, Zheng TT, Zhou CZ, Wan XH. 2022. Hyperoside: a review on its sources, biological activities, and molecular mechanisms. Phytother Res 36:2779–2802. doi:10.1002/ptr.7478 35561084

[8] Wu W, Xie Z, Zhang Q, Ma Y, Bi X, Yang X, Li B, Chen J. 2020. Hyperoside ameliorates diabetic retinopathy via anti-oxidation, inhibiting cell damage and apoptosis induced by high glucose. Front Pharmacol 11:797. doi:10.3389/fphar.2020.00797 32547397 PMC7273924

[9] Jang E. 2022. Hyperoside as a potential natural product targeting oxidative stress in liver diseases. Antioxidants (Basel) 11:1437. doi:10.3390/antiox11081437 35892639 PMC9331122

[10] Wang J, Sun H, Su M, Li Z, Li L, Zhao F, Zhang Y, Bai W, Yu S, Yang X, Qi S, Yang D, Guo D, Li C, Zhu Q, Xing X, Sun D. 2024. Natural hyperoside extracted from hawthorn exhibits antiviral activity against porcine epidemic diarrhea virus in vitro and in vivo. Virology (Auckl) 594:110037. doi:10.1016/j.virol.2024.110037 38498965

[11] Qiu J, Zhang T, Zhu X, Yang C, Wang Y, Zhou N, Ju B, Zhou T, Deng G, Qiu C. 2019. Hyperoside induces breast cancer cells apoptosis via ROS-mediated NF-κB signaling pathway. IJMS 21:131. doi:10.3390/ijms21010131 31878204 PMC6981893

[12] Fritz D, Venturi CR, Cargnin S, Schripsema J, Roehe PM, Montanha JA, von Poser GL. 2007. Herpes virus inhibitory substances from Hypericum connatum Lam., a plant used in southern Brazil to treat oral lesions. J Ethnopharmacol 113:517–520. doi:10.1016/j.jep.2007.07.013 17719731

[13] Shen B, Wu N, Shen C, Zhang F, Wu Y, Xu P, Zhang L, Wu W, Lu Y, Han J, Wang Y, Yuan H. 2016. Hyperoside nanocrystals for HBV treatment: process optimization, in vitro and in vivo evaluation. Drug Dev Ind Pharm 42:1772–1781. doi:10.3109/03639045.2016.1173051 27032257

[14] Chen H, Muhammad I, Zhang Y, Ren Y, Zhang R, Huang X, Diao L, Liu H, Li X, Sun X, Abbas G, Li G. 2019. Antiviral activity against infectious bronchitis virus and bioactive components of Hypericum perforatum L. Front Pharmacol 10:1272. doi:10.3389/fphar.2019.01272 31736754 PMC6830131

[15] El-Shiekh RA, Ashour RMS, Okba MM, Mandour AA, Kutkat O, Moatasim Y, Elshimy R. 2024. Natural compounds as possible anti-SARS-CoV-2 therapeutic agents: an in-vitro and in-silico study. Nat Prod Res 38:3836–3841. doi:10.1080/14786419.2023.2261069 37752734

[16] Pandarangga P, Simarmata YTRMR, Liu ABH, Haryati DAF. 2024. In silico simulation of hyperoside, isoquercetin, quercetin, and quercitrin as potential antivirals against the pNP868R protein of African swine fever virus. Vet World 17:171–178. doi:10.14202/vetworld.2024.171-178 38406373 PMC10884570

[17] Wang T, Hu L, Li R, Ren H, Li S, Sun Q, Ding X, Li Y, Wang C, Li L. 2024. Hyperoside inhibits EHV-8 infection via alleviating oxidative stress and IFN production through activating JNK/Keap1/Nrf2/HO-1 signaling pathways. J Virol 98:e0015924. doi:10.1128/jvi.00159-24 38499512 PMC11019850

[18] Lannes N, Summerfield A, Filgueira L. 2017. Regulation of inflammation in Japanese encephalitis. J Neuroinflammation 14:158. doi:10.1186/s12974-017-0931-5 28807053 PMC5557552

[19] Yu ZQ, Yi HY, Ma J, Wei YF, Cai MK, Li Q, Qin CX, Chen YJ, Han XL, Zhong RT, Chen Y, Liang G, Deng Q, Tian K, Wang H, Zhang GH. 2019. Ginsenoside Rg1 suppresses type 2 PRRSV infection via NF-κB signaling pathway in vitro, and provides partial protection against HP-PRRSV in piglet. Viruses 11:1045. doi:10.3390/v11111045 31717616 PMC6893584

[20] Huang B, Li F, You D, Deng L, Xu T, Lai S, Ai Y, Huang J, Zhou Y, Ge L, Zeng X, Xu Z, Zhu L. 2024. Porcine reproductive and respiratory syndrome virus infects the reproductive system of male piglets and impairs development of the blood-testis barrier. Virulence 15:2384564. doi:10.1080/21505594.2024.2384564 39072452 PMC11290757

[21] Zhai Y, Du Y, Yuan H, Fan S, Chen X, Wang J, He W, Han S, Zhang Y, Hu M, Zhang G, Kong Z, Wan B. 2024. Ubiquitin-specific proteinase 1 stabilizes PRRSV nonstructural protein Nsp1β to promote viral replication by regulating K48 ubiquitination. J Virol 98:e0168623. doi:10.1128/jvi.01686-23 38376196 PMC10949481

[22] Hu J, Li C, Zhou Y, Ding J, Li X, Li Y. 2023. Allicin inhibits porcine reproductive and respiratory syndrome virus infection in vitro and alleviates inflammatory responses. Viruses 15:1050. doi:10.3390/v15051050 37243135 PMC10220932

[23] Pang Y, Wang Y, Li C, Liu J, Duan C, Zhou Y, Fang L, Xiao S. 2022. Itaconate derivative 4-OI inhibits PRRSV proliferation and associated inflammatory response. Virology (Auckl) 577:84–90. doi:10.1016/j.virol.2022.10.007 36323047

[24] Heinz J, Kennedy PGE, Mogensen TH. 2021. The role of autophagy in varicella zoster virus infection. Viruses 13:1053. doi:10.3390/v13061053 34199543 PMC8227580

[25] Liu S, Yao S, Yang H, Liu S, Wang Y. 2023. Autophagy: regulator of cell death. Cell Death Dis 14:648. doi:10.1038/s41419-023-06154-8 37794028 PMC10551038

[26] Li S, Xu B, Luo Y, Luo J, Huang S, Guo X. 2024. Autophagy and apoptosis in rabies virus replication. Cells 13:183. doi:10.3390/cells13020183 38247875 PMC10814280

[27] Zhu X, Guan Z, Fang Y, Zhang Y, Guan Z, Li S, Peng K. 2023. Rift valley fever virus nucleoprotein triggers autophagy to dampen antiviral innate immune responses. J Virol 97:e0181422. doi:10.1128/jvi.01814-22 36939341 PMC10134837

[28] Hou P, Wang X, Wang H, Wang T, Yu Z, Xu C, Zhao Y, Wang W, Zhao Y, Chu F, Chang H, Zhu H, Lu J, Zhang F, Liang X, Li X, Wang S, Gao Y, He H. 2023. The ORF7a protein of SARS-CoV-2 initiates autophagy and limits autophagosome-lysosome fusion via degradation of SNAP29 to promote virus replication. Autophagy 19:551–569. doi:10.1080/15548627.2022.2084686 35670302 PMC9851267

[29] Zhao S, Qian Q, Chen X, Lu Q, Xing G, Qiao S, Li R, Zhang G. 2024. Porcine reproductive and respiratory syndrome virus triggers golgi apparatus fragmentation-mediated autophagy to facilitate viral self-replication. J Virol 98:e0184223. doi:10.1128/jvi.01842-23 38179942 PMC10878038

[30] Jiang C, Diao F, Ma Z, Zhang J, Bai J, Nauwynck H, Jiang P, Liu X. 2023. Autophagy induced by Rab1a-ULK1 interaction promotes porcine reproductive and respiratory syndrome virus replication. Virus Res 323:198989. doi:10.1016/j.virusres.2022.198989 36306941 PMC10194350

[31] Sun N, Sun P, Yao M, Khan A, Sun Y, Fan K, Yin W, Li H. 2019. Autophagy involved in antiviral activity of sodium tanshinone IIA sulfonate against porcine reproductive and respiratory syndrome virus infection in vitro*.* Antivir Ther 24:27–33. doi:10.3851/IMP3268 30272564

[32] Fan HH, Zhu LB, Li T, Zhu H, Wang YN, Ren XL, Hu BL, Huang CP, Zhu JH, Zhang X. 2017. Hyperoside inhibits lipopolysaccharide-induced inflammatory responses in microglial cells via p38 and NFκB pathways. Int Immunopharmacol 50:14–21. doi:10.1016/j.intimp.2017.06.004 28622577

[33] Fan H, Li Y, Sun M, Xiao W, Song L, Wang Q, Zhang B, Yu J, Jin X, Ma C, Chai Z. 2021. Hyperoside reduces rotenone-induced neuronal injury by suppressing autophagy. Neurochem Res 46:3149–3158. doi:10.1007/s11064-021-03404-z 34415495

[34] Mai J, He Q, Liu Y, Hou Y. 2023. Hyperoside attenuates sepsis-induced acute lung injury (ALI) through autophagy regulation and inflammation suppression. Mediators Inflamm 2023:1257615. doi:10.1155/2023/1257615 37545738 PMC10400302

[35] Li L, Xue B, Sun W, Gu G, Hou G, Zhang L, Wu C, Zhao Q, Zhang Y, Zhang G, Hiscox JA, Nan Y, Zhou EM. 2018. Recombinant MYH9 protein C-terminal domain blocks porcine reproductive and respiratory syndrome virus internalization by direct interaction with viral glycoprotein 5. Antiviral Res 156:10–20. doi:10.1016/j.antiviral.2018.06.001 29879459

[36] Li L, Wang J, Chen L, Ren Q, Akhtar MF, Liu W, Wang C, Cao S, Liu W, Zhao Q, Li Y, Wang T. 2024. Diltiazem HCl suppresses porcine reproductive and respiratory syndrome virus infection in susceptible cells and in swine. Vet Microbiol 292:110054. doi:10.1016/j.vetmic.2024.110054 38507832

[37] Wang T, Li S, Hu X, Geng Y, Chen L, Liu W, Zhao J, Tian W, Wang C, Li Y, Li L. 2024. Heme oxygenase-1 is an equid alphaherpesvirus 8 replication restriction host protein and suppresses viral replication via the PKCβ/ERK1/ERK2 and NO/cGMP/PKG pathway. Microbiol Spectr 12:e0322023. doi:10.1128/spectrum.03220-23 38441979 PMC10986571

[38] Wang T, Du Q, Niu Y, Zhang X, Wang Z, Wu X, Yang X, Zhao X, Liu SL, Tong D, Huang Y. 2019. Cellular p32 is a critical regulator of porcine circovirus type 2 nuclear egress. J Virol 93:e00979-19. doi:10.1128/JVI.00979-19 31511386 PMC6854514

[39] Wang T, Du Q, Wu X, Niu Y, Guan L, Wang Z, Zhao X, Liu SL, Tong D, Huang Y. 2018. Porcine MKRN1 modulates the replication and pathogenesis of porcine circovirus type 2 by inducing capsid protein ubiquitination and degradation. J Virol 92:e00100-18. doi:10.1128/JVI.00100-18 29514908 PMC5952126

[40] Diao F, Jiang C, Sun Y, Gao Y, Bai J, Nauwynck H, Wang X, Yang Y, Jiang P, Liu X. 2023. Porcine reproductive and respiratory syndrome virus infection triggers autophagy via ER stress-induced calcium signaling to facilitate virus replication. PLoS Pathog 19:e1011295. doi:10.1371/journal.ppat.1011295 36972295 PMC10079224

[41] Liu X, Bai J, Jiang C, Song Z, Zhao Y, Nauwynck H, Jiang P. 2019. Therapeutic effect of Xanthohumol against highly pathogenic porcine reproductive and respiratory syndrome viruses. Vet Microbiol 238:108431. doi:10.1016/j.vetmic.2019.108431 31648725

[42] Liu X, Song Z, Bai J, Nauwynck H, Zhao Y, Jiang P. 2019. Xanthohumol inhibits PRRSV proliferation and alleviates oxidative stress induced by PRRSV via the Nrf2-HMOX1 axis. Vet Res 50:61. doi:10.1186/s13567-019-0679-2 31506103 PMC6737628

[43] Wang HM, Liu TX, Wang TY, Wang G, Liu YG, Liu SG, Tang YD, Cai XH. 2018. Isobavachalcone inhibits post-entry stages of the porcine reproductive and respiratory syndrome virus life cycle. Arch Virol 163:1263–1270. doi:10.1007/s00705-018-3755-4 29411137 PMC7086980

[44] Hao H, Wen L, Li J, Wang Y, Ni B, Wang R, Wang X, Sun M, Fan H, Mao X. 2015. LiCl inhibits PRRSV infection by enhancing Wnt/β-catenin pathway and suppressing inflammatory responses. Antiviral Res 117:99–109. doi:10.1016/j.antiviral.2015.02.010 25746333

[45] Ahn H, Lee GS. 2017. Isorhamnetin and hyperoside derived from water dropwort inhibits inflammasome activation. Phytomedicine 24:77–86. doi:10.1016/j.phymed.2016.11.019 28160865

[46] Yu C, Wang D, Yang Z, Wang T. 2022. Pharmacological effects of polyphenol phytochemicals on the intestinal inflammation via targeting TLR4/NF-κB signaling pathway. IJMS 23:6939. doi:10.3390/ijms23136939 35805952 PMC9266441

[47] Kuzmich NN, Sivak KV, Chubarev VN, Porozov YB, Savateeva-Lyubimova TN, Peri F. 2017. TLR4 signaling pathway modulators as potential therapeutics in inflammation and sepsis. Vaccines (Basel) 5:34. doi:10.3390/vaccines5040034 28976923 PMC5748601

[48] Liu F, Zhao Y, Pei Y, Lian F, Lin H. 2024. Role of the NF-kB signalling pathway in heterotopic ossification: biological and therapeutic significance. Cell Commun Signal 22:159. doi:10.1186/s12964-024-01533-w 38439078 PMC10910758

[49] Sun MX, Huang L, Wang R, Yu YL, Li C, Li PP, Hu XC, Hao HP, Ishag HA, Mao X. 2012. Porcine reproductive and respiratory syndrome virus induces autophagy to promote virus replication. Autophagy 8:1434–1447. doi:10.4161/auto.21159 22739997

[50] Rathore AS, Singh SS, Birla H, Zahra W, Keshri PK, Dilnashin H, Singh R, Singh S, Singh SP. 2023. Curcumin modulates p62-Keap1-Nrf2-mediated autophagy in rotenone-induced parkinson’s disease mouse models. ACS Chem Neurosci. doi:10.1021/acschemneuro.2c00706 36989171

[51] Kong L, Deng J, Zhou X, Cai B, Zhang B, Chen X, Chen Z, Wang W. 2021. Sitagliptin activates the p62-Keap1-Nrf2 signalling pathway to alleviate oxidative stress and excessive autophagy in severe acute pancreatitis-related acute lung injury. Cell Death Dis 12:928. doi:10.1038/s41419-021-04227-0 34635643 PMC8505515

[52] Chen J, Zhang J, Chen T, Bao S, Li J, Wei H, Hu X, Liang Y, Liu F, Yan S. 2022. Xiaojianzhong decoction attenuates gastric mucosal injury by activating the p62/Keap1/Nrf2 signaling pathway to inhibit ferroptosis. Biomedicine & Pharmacotherapy 155:113631. doi:10.1016/j.biopha.2022.113631 36122518

[53] Cheng J, Xu J, Gu Y, Wang Y, Wang J, Sun F. 2024. Melatonin ameliorates 10-hydroxycamptothecin-induced oxidative stress and apoptosis via autophagy-regulated p62/Keap1/Nrf2 pathway in mouse testicular cells. J Pineal Res 76:e12959. doi:10.1111/jpi.12959 38738543

[54] Avula K, Singh B, Kumar PV, Syed GH. 2021. Role of lipid transfer proteins (LTPs) in the viral life cycle. Front Microbiol 12:673509. doi:10.3389/fmicb.2021.673509 34248884 PMC8260984

[55] Yeh Y-C, Doan LH, Huang Z-Y, Chu L-W, Shi T-H, Lee Y-R, Wu C-T, Lin C-H, Chiang S-T, Liu H-K, Chuang T-H, Ping Y-H, Liu H-S, Huang C-YF. 2021. Honeysuckle (Lonicera japonica) and Huangqi (Astragalus membranaceus) Suppress SARS-CoV-2 entry and COVID-19 related cytokine storm in vitro. Front Pharmacol 12:765553. doi:10.3389/fphar.2021.765553 35401158 PMC8990830

[56] Wei J, Ma Y, Wang L, Chi X, Yan R, Wang S, Li X, Chen X, Shao W, Chen JL. 2017. Alpha/beta interferon receptor deficiency in mice significantly enhances susceptibility of the animals to pseudorabies virus infection. Vet Microbiol 203:234–244. doi:10.1016/j.vetmic.2017.03.022 28619150

[57] Yang L, Wen J, Zhang Y, Liu Z, Luo Z, Xu L, Lai S, Tang H, Sun X, Hu Y, Zhu L, Xu Z. 2022. The antiviral activity of caprylic monoglyceride against porcine reproductive and respiratory syndrome virus in vitro and in vivo. Molecules 27:7263. doi:10.3390/molecules27217263 36364088 PMC9653991

[58] Schlett JS, Mettang M, Skaf A, Schweizer P, Errerd A, Mulugeta EA, Hein TM, Tsesmelis K, Tsesmelis M, Büttner UFG, Wendt H, Abaei A, Rasche V, Prex V, Nespoli E, Alami NO, Tews D, Walther P, Yilmazer-Hanke D, Oswald F, Dimou L, Wirth T, Baumann B. 2023. NF-κB is a critical mediator of post-mitotic senescence in oligodendrocytes and subsequent white matter loss. Mol Neurodegener 18:24. doi:10.1186/s13024-023-00616-5 37069623 PMC10108549

[59] Xu Y, Wang H, Zhang X, Zheng X, Zhu Y, Han H, Feng WH. 2021. Highly pathogenic porcine reproductive and respiratory syndrome virus (HP-PRRSV) induces IL-6 production through TAK-1/JNK/AP-1 and TAK-1/NF-κB signaling pathways. Vet Microbiol 256:109061. doi:10.1016/j.vetmic.2021.109061 33836390

[60] Kim SJ, Um JY, Lee JY. 2011. Anti-inflammatory activity of hyperoside through the suppression of nuclear factor-κB activation in mouse peritoneal macrophages. Am J Chin Med 39:171–181. doi:10.1142/S0192415X11008737 21213407

[61] Chen T, Tu S, Ding L, Jin M, Chen H, Zhou H. 2023. The role of autophagy in viral infections. J Biomed Sci 30:5. doi:10.1186/s12929-023-00899-2 36653801 PMC9846652

