# Supplementary material for: Hyperoside inhibits PRRSV proliferation via the TLR4/NF-κB and p62-Nrf2-Keap1 signaling pathways, mediating inflammation and autophagy
Source: Microbiol Spectr. 2025 Jun 12;13(8):e03107-24. doi: 10.1128/spectrum.03107-24 (PMC12323607; doi:10.1128/spectrum.03107-24)
Supplement: Supplemental material — Fig. S1 to S4. [file spectrum.03107-24-s0001.docx]

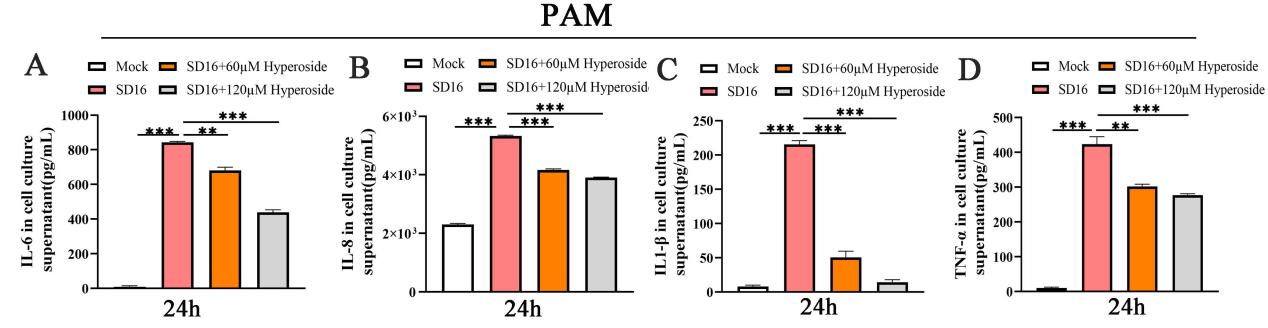


**Figure S1.** Hyperoside alleviates the proinflammatory response in infected PAMs.

PAMs were seeded into 12-well plates and infected with PRRSV SD16 (MOI = 0.1) in the presence of hyperoside (120 µM) or 0.1% DMSO. The supernatants were harvested at 24 hpi, and the production of IL-6 (A), IL-8 (B), IL-1β (C), and TNF-α (D) was analyzed via ELISA. **, *P*< 0.01; ***, *P*< 0.001.


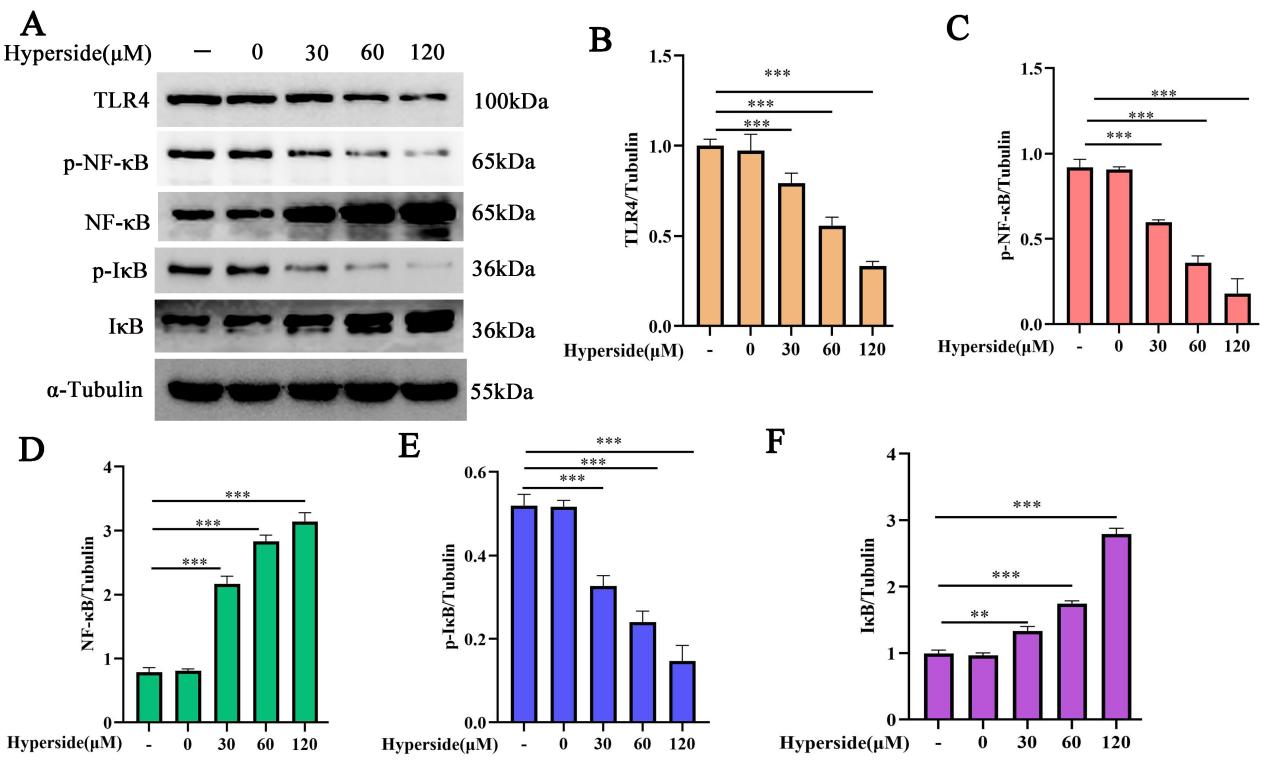


**Figure S2. Hyperoside regulates the expression of the TLR4/**NF-κB signaling pathway.

MARC-145 cells were added to 12-well plates and incubated with different concentrations of hyperoside (30, 60 and 120 µM) for 24 h. The cells were collected, and TLR4, p-NF-κB p65, NF-κB p65, p-IκBα, and IκBα protein expression was detected via western blotting (A). The quantitative analysis of TLR4 (B), p-NF-κB p65 (C), NF-κB p65 (D), p-IκBα (E), and IκBα (F) was conducted via ImageJ. The values are presented as the means ± SDs. **, *P*<0.01; ***, *P*<0.001.


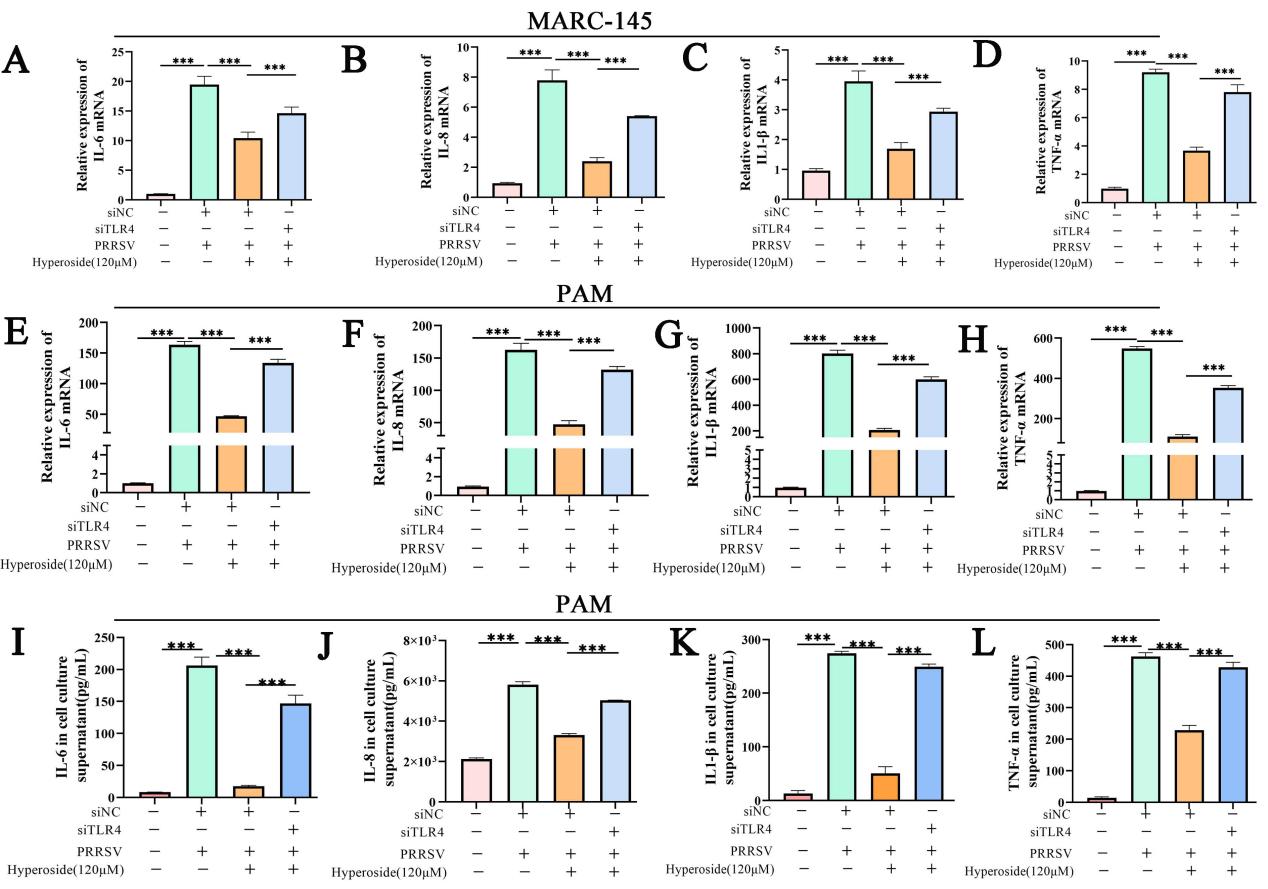


**Figure S3.** Hyperoside alleviates the proinflammatory response through TLR4 in infected susceptible cells.

MARC-145 cells or PAMs were seeded into 12-well plates, transfected with siTLR4 (100 µM) for 12 h, and then infected with PRRSV SD16 (MOI = 0.1) in the presence of hyperoside (120 µM). MARC-145 (A-D) or PAMs (E-H) were harvested at 24 hpi, and the mRNA levels of IL-6, IL-8, IL-1β and TNF-α were measured via qPCR. Supernatants of PAMs were collected at 24 hpi, and the production of IL-6 (I), IL-8 (J), IL-1β (K), and TNF-α (L) was detected via ELISA. ***, *P*< 0.001.


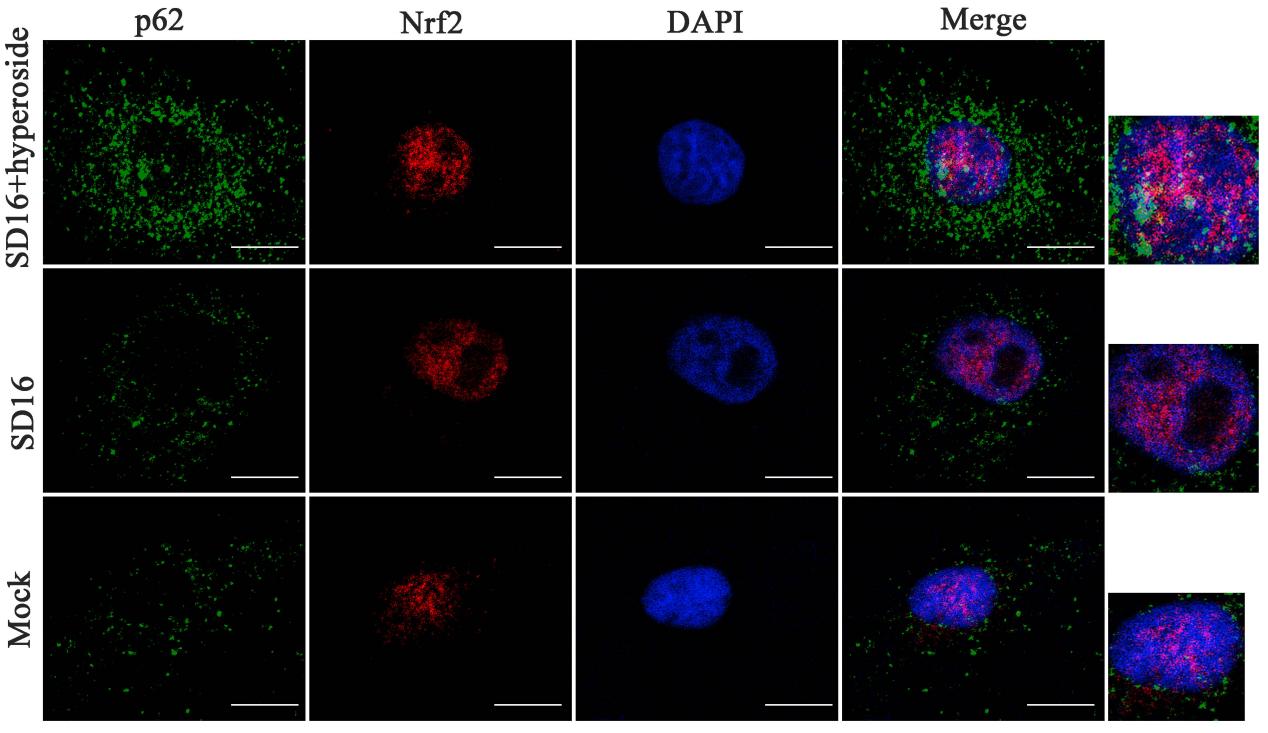


**Figure S4. Nrf2 binding to p62 in the nucleus of hyperoside-treated MARC-145 cells.**

MARC-145 cells were pretreated with hyperoside (120 μM) for 2 h and then infected with PRRSV SD16 (MOI = 0.5) for 1 h. After 24 h, these cells were fixed to analyze the localization of the autophagy-related proteins p62 and Nrf2 via confocal microscopy. Scale bar 10 μm.
